# Fish Oil and Selenium with Doxorubicin Modulates Expression of Fatty Acid Receptors and Selenoproteins, and Targets Multiple Anti-Cancer Signaling in Triple-negative Breast Cancer Tumors

**DOI:** 10.7150/ijms.75848

**Published:** 2022-11-14

**Authors:** Chih-Hung Guo, Min-Yi Shih, Chieh-Han Chung, Yi-Chun Lin, Ciou-Ting Fan, Chia-Lin Peng, Pei-Chung Chen, Simon Hsia

**Affiliations:** 1 Micronutrition and Biomedical Nutrition Labs, Institute of Biomedical Nutrition, Hung-Kuang University, Taichung 433, Taiwan; 2 Taiwan Nutraceutical Association, Taipei 105, Taiwan

**Keywords:** Anticancer signaling, Doxorubicin, Fish oil and selenium, GPR-40, Immune microenvironment, Selenoproteins, TNBC mouse tumor

## Abstract

Omega-3 fatty acids from fish oil (FO) and selenium (Se) potentiate some conventional therapies and have anticancer immune potential. This study aims to determine whether FO/Se modulates G-protein-coupled polyunsaturated fatty acid receptors (GPR-40 and GPR-120) and selenoproteins (Sel-H, Sel-W, and GPx4), and increases the therapeutic effect of doxorubicin in a dose-dependent manner on triple-negative breast cancer (TNBC) mouse. Mice were randomized into 5 groups (n = 7/group) and treated with physiological saline (control), low-dose doxorubicin, and doxorubicin in combination with low, medium, or high doses of FO/Se. The expression of signaling molecules in tumors was determined by measuring either mRNA or protein expression. Compared with doxorubicin alone, combination treatment resulted in lower tumor sizes and fewer overall metastasis, lower GPR-40 mRNA levels, and higher expression of all selenoproteins. Doxorubicin-FO/Se combination treatment decreased expression of membrane EGFR and FGFR, down-regulated downstream PI3K/AKT/mTOR, MAPK/ERK, and JAK2/c-Src/STAT3 signaling, increased tumor suppressor PTEN/TSC1/TSC2 expression and P53 activation, and suppressed oncogenic transcription factor expression. Dose-dependent inhibition of proliferation index Ki-67, cell cycle, and stem-cell-related markers were observed. Decreased immune check-points PD-L1/CTLA-4/Foxp3/CD86 and increased PD-1/CD28/IL-2 expression was also found. These observations suggest that the nutritional supplements FO/Se increase the chemotherapeutic efficacy of doxorubicin against TNBC by modulating GPR-40 and selenoprotein and targeting multiple signaling pathways in tumor tissues.

## Introduction

Breast cancer is the most frequently diagnosed cancer in women worldwide. Triple-negative breast cancer (TNBC) is characterized by its lack of expression of estrogen receptors, progesterone receptors, and human epidermal growth factor receptor 2 1. TNBC patients have a higher incidence of invasive and metastatic cancer, resistance to chemotherapy, earlier recurrence, and shorter overall survival than do patients with other types of breast cancer. Targeted therapy is unavailable, leaving only conventional chemotherapies such as with doxorubicin (adriamycin), taxanes (docetaxel and paclitaxel), and platinum agents 1,2.

Doxorubicin is widely used to treat TNBC. This drug directly induces DNA oxidative damage and AMP-activated protein kinase-related apoptosis and inactivates the Src-dependent epidermal growth factor receptor (EGFR) signaling pathway 3,4. However, doxorubicin produces severe adverse events and, in time, most TNBC patients experience distant relapse 5. Reducing the doxorubicin dosage can decrease side effects, but at the expense of anti-cancer efficacy. In addition to these drawbacks, the development of doxorubicin resistance is common. Such resistance results from the overactivation of mitogen-activated protein kinase/extracellular signal-regulated kinase (MAPK/ERK) and phosphati-dylinositol 3-kinase/protein kinase B/mammalian target of rapamycin (PI3K/Akt/mTOR) pathways by EGFR aberration 6. Therefore, novel strategies are needed to increase the anti-tumor efficacy of doxo-rubicin at low doses and overcome doxorubicin resistance.

Promising new targets for anti-TNBC treatment include the fibroblast growth factor receptor (FGFR), trophoblast cell-surface antigen 2 (Trop-2), and membrane-associated MAPK/ERK, PI3K/Akt/mTOR, and Janus kinase 2/signal transducer and activator of transcription 3 (JAK2/STAT3) signaling pathways, and hypoxia-inducible factor (HIF)-1α suppression 7,8. Furthermore, activation of JAK2/ STAT3, PI3K/Akt/mTOR, and MAPK/ERK pathways, nuclear factor kappa-light-chain-enhancer of activated B cells (NF-κB), and HIF-1α can regulates the programmed cell death 1 (PD-1)/programmed cell death-ligand (PD-L1/2) immune signaling pathway in various types of cancer cells 9. Accumulating evidence indicates that tumor immune microenvironment (particularly regarding PD-1, PD-L1/2, forkhead box P3 (Foxp3), CD80, and CD86 expression) and immune checkpoint signaling play decisive roles in tumor immune evasion and growth 10. Thus, immunotherapeutic strategies are of great interest for treating TNBC. Antibodies against cytotoxic T-lymphocyte-associated protein-4 (CTLA-4), PD-1, and PD-L1 have been evaluated in several clinical trials; whereas these studies included only a subset of TNBC patients, and adverse reactions occurred frequently 11. It is suggested that downregulate signaling pathways that drive tumor progression while target the immune microenvironment will be an important therapeutic approach to TNBC treatment.

Fish oil (FO) containing eicosapentaenoic acid (EPA, 20:5n-3) and docosahexaenoic acid (DHA, 22:6n-3), and essential micronutrient selenium (Se) play crucial roles in anti-cancer therapy of experimental models. Recent studies have reported synergistic effects of nutritional supplementation with FO and Se on apoptosis and reduced resistance to EGFR inhibitors in some cancer cells 12,13. We previously reported that combination treatment with a low-dose FO/Se shows potential as a possible adjuvant therapy to modulate Th1/Th2-related cytokine production and decrease AXL receptor tyrosine kinase, heat shock proteins, matrix metalloproteinase-9, HIF-1α, and PD-L1 protein levels in a TNBC murine model treatment with either bevacizumab (avastin) or doxorubicin 14. Furthermore, combination treatments with bevacizumab plus FO/Se have been shown to significantly inhibit tumor PI3K/Akt/mTOR and Ras/Raf/MEK/ERK signaling, and anti-apoptotic proteins in a dose-dependent manner by FO/Se in TNBC tumor-bearing mice 15. Thus, the combination treatment with doxorubicin and high-dose FO/Se may be safe and more efficacious for treating TNBC than does doxorubicin combined with low-dose FO/Se.

The underlying mechanism of FO/Se are not yet clear and limited studies have evaluated potential toxicity of combination treatment with doxorubicin and FO/Se. We therefore hypothesize that FO/Se exerts its anti-cancer effect directly by influencing G-protein-coupled polyunsaturated fatty acid receptors (GPR) and Se-containing selenoproteins (Sels) in TNBC tumor. This study aims to investigate the modulation of GPR-40/-120 and Sels, dual EGFR and FGFR inhibition, and alteration in the downstream signaling targets and immune microenvironment by combining doxorubicin with three doses of FO/Se in mice with triple-negative 4T1 mammary adenocarcinoma. Selected plasma bio-chemical indices of liver and renal function are also determined.

## Materials and Methods

### Materials

The murine 4T1 breast carcinoma cell line CRL-2539 spontaneously produces highly metastatic tumors. This cell line was obtained from the American Type Culture Collection (Rockville, MD, USA) and maintained in RPMI 1640 medium (Gibco BRL, Gaithersburg, MD, USA) supplemented with 10% (v/v) heat-inactivated fetal bovine serum and 1% penicillin-streptomycin at 37°C in a humidified atmosphere with 5% CO_2_. Doxorubicin (Pfizer New Zealand Ltd., Auckland, New Zealand) was purchased from Sigma-Aldrich (St. Louis, MO, USA). As described previously 15, FO- and Se-free powdered medium (control) was purchased from Do Well Laboratories, Inc. (Irvine, CA, USA). Supplemental FO and Se from Se yeast complex (Do Well Laboratories) were mixed with the control powder to provide low, medium, and high doses of FO/elemental Se, as follows: low, 8.8 mg/2.7 μg/g; medium, 16.9 mg/4.0 μg/g; and high, 19.0 mg/6.7 μg/g, respectively.

### Animals and tumor implantation

Animal experiments were conducted in compliance with procedures approved by the Institutional Animal Care Committee of Hung Kuang University. Seven-week-old female BALB/cByJNarl mice were purchased from the National Animal Laboratory Breeding Research Centre (Nangang District, Taipei City, Taiwan). All animals were managed on a controlled 12-hr light/dark cycle at 24 ± 1°C and 60-70% relative humidity and were given rodent chow (Ralston Purina, Lab Diet #5001, St. Louis, MO, USA) and distilled deionized water ad libitum throughout the study.

The heterotopic model is widely used in breast cancer research 16. In the present study, mice were allowed an acclimatization period of one week before the experiments began. Mouse 4T1 tumor cells (1×10^5^) were subcutaneously implanted in the mouse right hind thigh on day 0 of the experiment. Mice bearing tumors on day 7 were randomized into 5 weight-matched groups of 7 mice each, as follows: Group 1 (control, no doxorubicin), injected with saline (0.9% NaCl); Group 2 (Doxo, doxorubicin group), injected intraperitoneally with 5 mg/kg doxorubicin (once every 4 days); Groups 3, 4, and 5 (low, medium, and high FO/Se group), injected intraperitoneally with 5 mg/kg doxorubicin (once every 4 days) together with 0.4 g of low, medium, and high concentrations of FO/Se by oral gavage twice a day from day 7 to day 31. The tumor size was estimated using the formula V = (X^2^Y)/2, where X and Y are the short and long diameters, respectively, as determined by caliper measurements. Tumor growth-curves were calculated from caliper measurements every 3 days. Additionally, non-tumor-bearing healthy mice were treated with and without 0.4 g of high dose-FO/Se (Healthy and H-high FO/Se groups) by oral gavage twice a day from day 7 to day 31. At the end of study day 32, all animals were sacrificed after blood collection; primary tumors and other tissues were carefully removed and weighed.

### Determination of alanine aminotransferase (ALT) and blood urea nitrogen (BUN)

The activity of enzyme ALT in blood was measured using commercially available diagnostic kits (Randox, Northern Ireland, UK). Additionally, the level of urea was determined using enzymatic kits (#ur446, Randox, Northern Ireland, UK) and BUN value was then calculated by dividing urea levels by 2.14.

### RNA isolation and real-time qPCR analysis

Total RNA from tumors in 3-4 mice per group was extracted using the Bio-Rad RNA kit (Bio-Rad Lab, Hercules, CA, USA), and RNA was then used for cDNA synthesis using a thermocycler (T100 Thermal Cycler) with the iScript cDNA synthesis kit (Bio-Rad Lab, Hercules, CA, USA). Briefly, amplified cDNA was assessed using the CFX Connect RT-PCR detection system with SYBR Green Supermix (Bio-Rad, Hercules, CA, USA). GAPDH was used for normalization. The primer sequences used for quantitative real-time PCR are shown in Supplementary Table S1. The expression data were directly normalized to GAPDH, and the fold-change was calculated using the threshold cycle (2-ΔΔCT) method.

### Western blot analysis

Tumor tissues from the remaining 3-4 mice in each group were lysed using ice-cold buffer containing 1% Nonidet P-40, 0.1% SDS, and 0.5% sodium deoxycholic acid, supplemented with a protease inhibitor cocktail (Biokit Biotech, Inc., Miaoli, Taiwan). Nuclear extracts were prepared using a cell nuclear protein extraction kit (Biokit Biotech) as described previously 15. Lysate protein concentrations were determined using the Bio-Rad Protein Assay (Bio-Rad), using a dilution series of bovine serum albumin as standards. Equal amounts of denatured proteins from every single animal were separated by SDS-PAGE, transferred to nitrocellulose membrane, incubated with different primary antibodies, then stored overnight at 4 °C. The membranes were washed with wash buffer, followed by incubation with horseradish peroxidase-conjugated goat anti-mouse IgG antibody at room temperature. Immuno-reactive bands were visualized using an enhanced chemiluminescence detection kit (PerkinElmer Life Sciences Inc., Waltham, MA, USA). Signal intensities were quantified using the Fujifilm LAS-4000 system and Multi Gauge 3.0 software (Fuji, Japan). Mouse primary antibodies used are described in Supplementary Table S2.

### Statistical analysis

Quantitative variables are presented as the mean (standard error). The level of statistical significance was set at p < 0.05. The Shapiro-Wilk test showed normal measurements, and Student's t-test and one-way ANOVA were used for comparisons, as appropriate. Duncan's multiple range test was used as a post-hoc test.

## Results

### Combination treatment with FO/Se and doxorubicin reduced tumor growth

**Figure 1:**
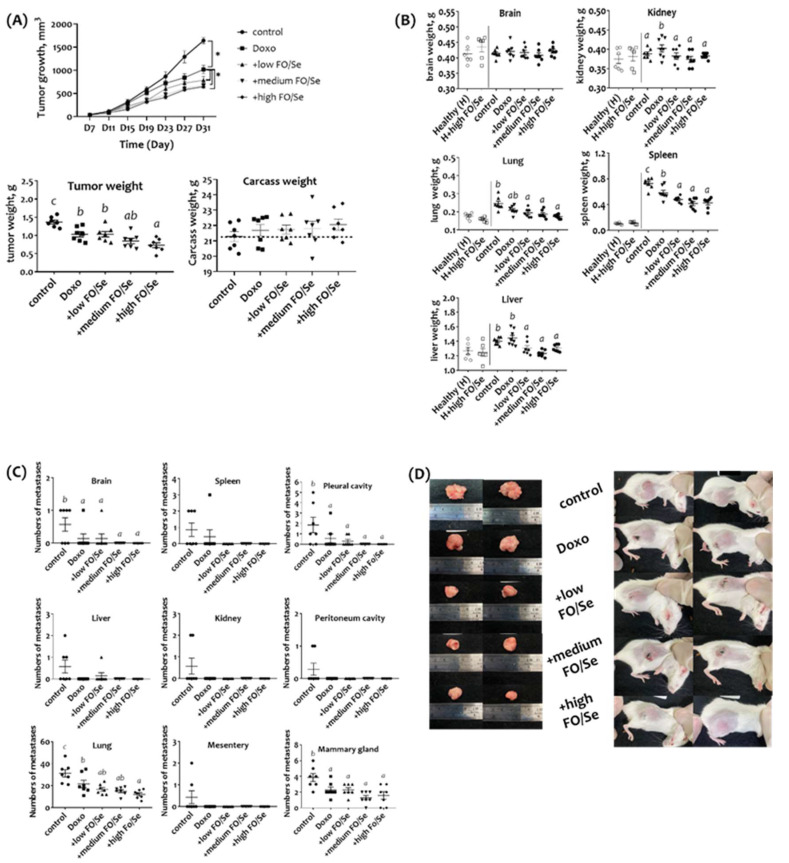
** (A) Tumor growth and tumor and body weights. (B) Internal organ weights. (C) Number of metastases in different organs. (D) Gross appearance of primary tumors in TNBC-bearing mice.** Values are expressed as the mean (standard error). *p < 0.05 doxorubicin vs control; doxorubicin vs + low, medium, and high FO/Se. 4T1 tumor cells (1 × 10^5^) were subcutaneously injected into the mouse right hind thigh on day 0. Tumor-bearing mice on day 7 were randomized into 5 weight-matched groups of 7 mice each as follows: 1) Control group, saline injected; 2) doxorubicin group, injected intraperitoneally with 5 mg/kg doxorubicin (once every 4 days); 3) +low FO/Se, +medium FO/Se, and +high FO/Se groups were injected intraperitoneally with 5 mg/kg doxorubicin (once every 4 days) together with a low, medium, or high dose of fish oil/selenium supplement p.o. twice a day from day 7 to day 31. Healthy mice were randomly assigned to Healthy (H, n=6) and H+high FO/Se (n=6) groups and treated with or without 0.4 g of high dose-FO/Se by oral gavage twice a day from day 7 to day 31. Doxo, doxorubicin; FO, fish oil; Se, selenium. Carcass weight = total body weight minus tumor weight. Superscript ^a, b, c, d^: Bars sharing the same superscript are not significantly different from each other; Bars with different superscript are significantly different from each other (*p* < 0.05).

A survival rate of 100% was observed in all groups (data not shown). Mice in the doxorubicin group had smaller tumors than did those in the control group; however, mice were observed biting the tumor tissue on day 27 in the doxorubicin group. Mice that received doxorubicin together with FO/Se had significantly smaller tumors than did those treated with doxorubicin alone (p < 0.05) (Figure 1A). The mean tumor size did not differ significantly between the 3 FO/Se groups. The mean tumor weight was significantly lower in the doxorubicin group than in the control group. The lowest tumor weight was observed in the high-FO/Se group. The body weights, subtracting the primary tumor weights, were non-significantly higher in mice treated with doxorubicin and FO/Se than in those receiving saline and doxorubicin alone.

### Combination treatment resulted in fewer metastases, and normalized organ weights and bio-chemical indices

No significant difference in serum levels of ALT and BUN between healthy mice treated with and without high dose of FO/Se (p > 0.05) (Supplementary Figure S1). There was no marked difference in the liver, kidney, lung, spleen, and brain weight between two groups (Figure 1B). Further, tumor-bearing mice in the control group had significantly higher levels of ALT and BUN than in the healthy group. In contrast, mice in the 3 FO/Se groups had lower levels of ALT and BUN than those in the doxorubicin group.

For tumor-bearing mice, kidney weights were significantly higher in the doxorubicin group than in the control group. Mice receiving combination treatment with doxorubicin and any concentration of FO/Se had lower-weight kidneys, lungs, spleen, and liver than those of the control and doxorubicin groups.

The mean counting of visible metastatic tumor nodules in each isolated tissues were determined by three laboratory technicians. Mice treated with doxorubicin alone or in combination with FO/Se had fewer brain, lung, pleural cavity, and mammary gland metastases than did control mice (Figure 1C). Mice in the high-FO/Se group had the lowest incidence of lung metastasis. Additionally, gross examination revealed that tumors in control mice were larger than those in mice treated with doxorubicin alone or in combination with FO/Se (Figure 1D).

### Combination treatment altered expression of selenoproteins and fatty acid receptors

**Figure 2:**
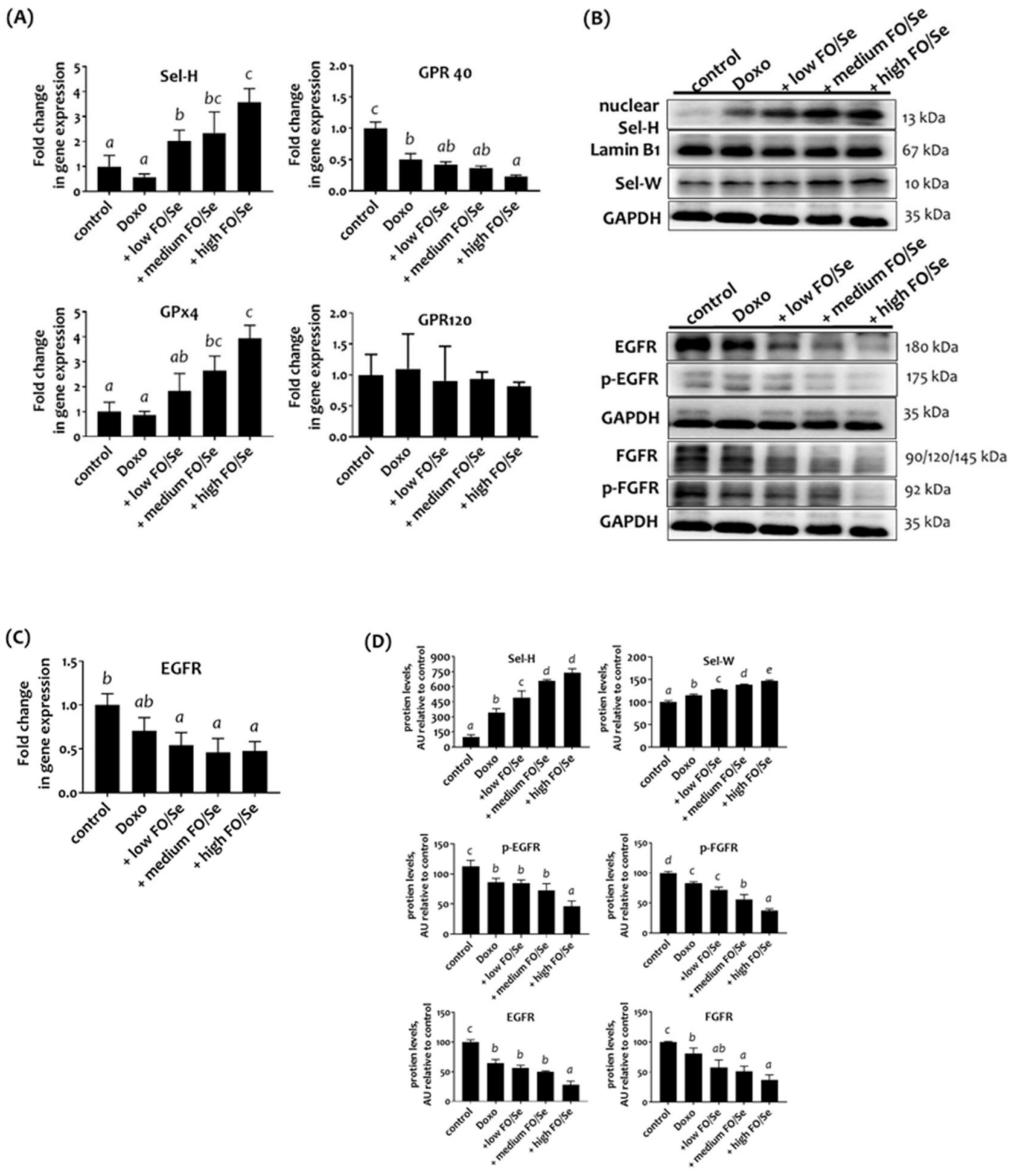
** (A) Selenoprotein (Sel-H and GPx4) and G-protein-coupled polyunsaturated fatty acid receptors (GPR-40/120) mRNA levels in TNBC mouse tumor. (B) Sel-H/Sel-W and EGFR/FGFR protein expression. (C) EGFR mRNA levels. (D) Densitometric analysis of three separate western blots.** Values are expressed as relative readings (mean ± standard error) from 3-4 mice in each group. Further details of the groups illustrated are described in Figure 1. Superscript ^a, b, c, d^: Bars sharing the same superscript are not significantly different from each other; Bars with different superscript are significantly different from each other (*p* < 0.05).

To determine the tumor content of Sels, the mRNA levels of Sel-H and glutathione peroxidase-4 (GPx4) were examined. No significant difference in the mRNA level of Sel-H or GPx4 was observed between the control and doxorubicin groups (Figure 2A). Mice that received doxorubicin together with FO/Se had higher mRNA levels than did those in the control and doxorubicin groups. As shown in Figure 2B, the tumors of mice treated with doxorubicin plus low-, medium-, or high-FO/Se had significantly higher expression of Sel-W and nuclear Sel-H protein than did those of control mice, with expression increasing in a dose-dependent manner.

Analysis of the expression of the G-protein-coupled polyunsaturated fatty acid receptors GRP-40 and GRP-120 showed that mice treated with doxorubicin alone or with FO/Se had lower mRNA levels of GPR-40 but not GPR-120 compared with control mice (Figure 2A). The lowest GPR-40 expression level was observed in the high FO/Se group.

### Combination treatment reduced expression of tumor receptor tyrosine kinases EGFR and FGFR

Mice treated with low-dose doxorubicin alone had non-significantly lower EGFR mRNA level in tumors than did the saline controls (Figure 2C). Tumors from doxorubicin-treated mice receiving FO/Se had much lower EGFR mRNA levels than did those from mice treated with doxorubicin alone. Further, tumors from doxorubicin-treated mice with and without FO/Se exhibited significantly lower expression of EGFR and phosphorylated (p)-EGFR proteins than did controls, with mice receiving high-dose FO/Se exhibiting the lowest tumor expression of these proteins (Figure 2D). Mice treated with doxorubicin alone exhibited lower protein expression levels of FGFR and p-FGFR in tumor tissues than did controls. The level of FGFR and FGFR phosphorylation in tumors was lower in mice treated with doxorubicin plus FO/Se than in those treated with doxorubicin alone.

### Combination treatment altered the tumor cytoplasmic signaling pathway

#### PI3K/PTEN/Akt/mTOR/TSC1,2/4EBP1/p70S6K axis

**Figure 3:**
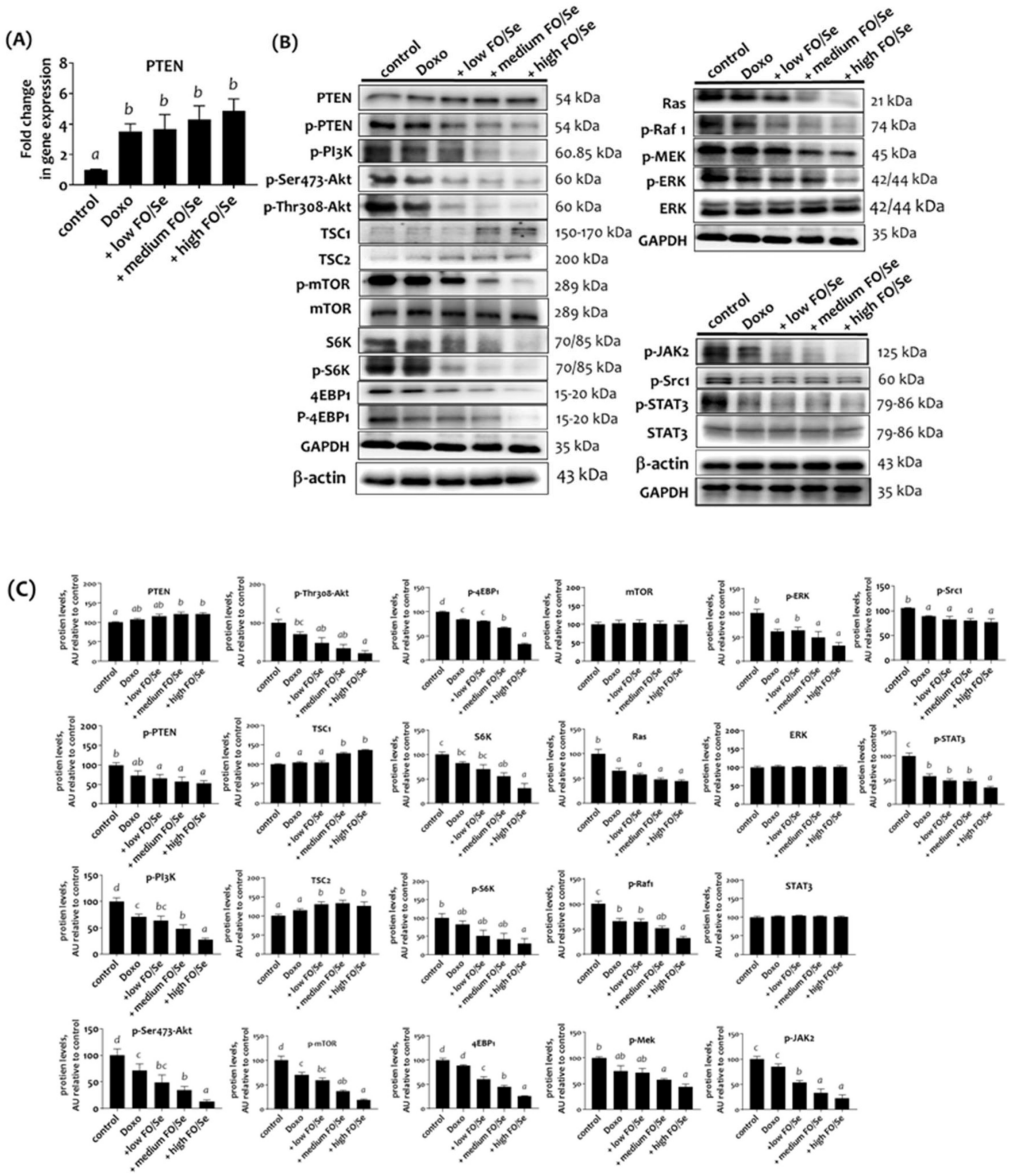
** (A) PTEN mRNA levels in TNBC mouse tumor. (B) Expression of PI3K/PTEN/AKT/mTOR/4EBP1/p70S6K, Ras/Raf/MEK/ERK, and c-Src/JAK2/STAT3 signaling markers. (C) Densitometric analysis of three separate western blots.** Values are expressed as relative readings (mean ± standard error) from 3-4 mice in each group. Further details of the groups illustrated are described in Figure 1. Superscript ^a, b, c, d^: Bars sharing the same superscript are not significantly different from each other; Bars with different superscript are significantly different from each other (*p* < 0.05).

Treatment with doxorubicin alone and in combination with FO/Se resulted in significantly higher mRNA levels of tumor suppressor PTEN than in controls (Figure 3A). Mice treated with doxorubicin alone exhibited non-significantly higher PTEN protein expression compared with controls. Further, combination treatment produced higher levels of PTEN protein (Figure 3B and 3C). Mice treated with doxorubicin had lower phosphorylated levels of PI3K, Akt, mTOR, eukaryotic translation initiation factor 4E- binding protein 1 (4EBP1), and ribosomal protein S6 kinase beta-1 (p70S6K) than did control mice. Combination treatment with doxorubicin and FO/Se further decreased these phosphorylated protein levels and increased the expression of tumor suppressor protein (tuberous sclerosis complex TSC1 and TSC2) compared to controls and treatment with doxorubicin alone.

#### Ras/Raf/MEK/ERK axis

Mice treated with doxorubicin alone exhibited lower tumor levels of Ras, phosphorylated-rapidly accelerated fibrosarcoma (p-Raf1), mitogen‑activated protein kinase kinase (p-MEK), and extracellular signal‑regulated kinase (p-ERK) than did controls (Figure 3B, 3C). Combined treatment with doxo-rubicin and FO/Se further decreased the levels of Ras, p-Raf1, p-MEK, and p-ERK1/2 proteins compared to tumors from mice treated with doxorubicin alone. A non-significant difference in total ERK levels was observed between the groups.

#### c-Src/JAK2/STAT3 axis

Doxorubicin-treated mice exhibited lower levels of phospho-c-Src and phopsho-JAK2 than did controls (Figure 3B and 3C), with the lowest levels observed in mice treated with doxorubicin plus FO/Se. Significantly lower levels of STAT3 phosphorylation were observed in mice treated with doxorubicin alone and combination treatment, with the high-FO/Se group exhibiting the lowest levels of p-STAT3 protein. Non-significant differences in STAT3 protein expression were observed between the groups.

### Combination treatment decreased the expression of tumor oncogenes and proteins

#### c-Jun and c-Fos mRNA

**Figure 4:**
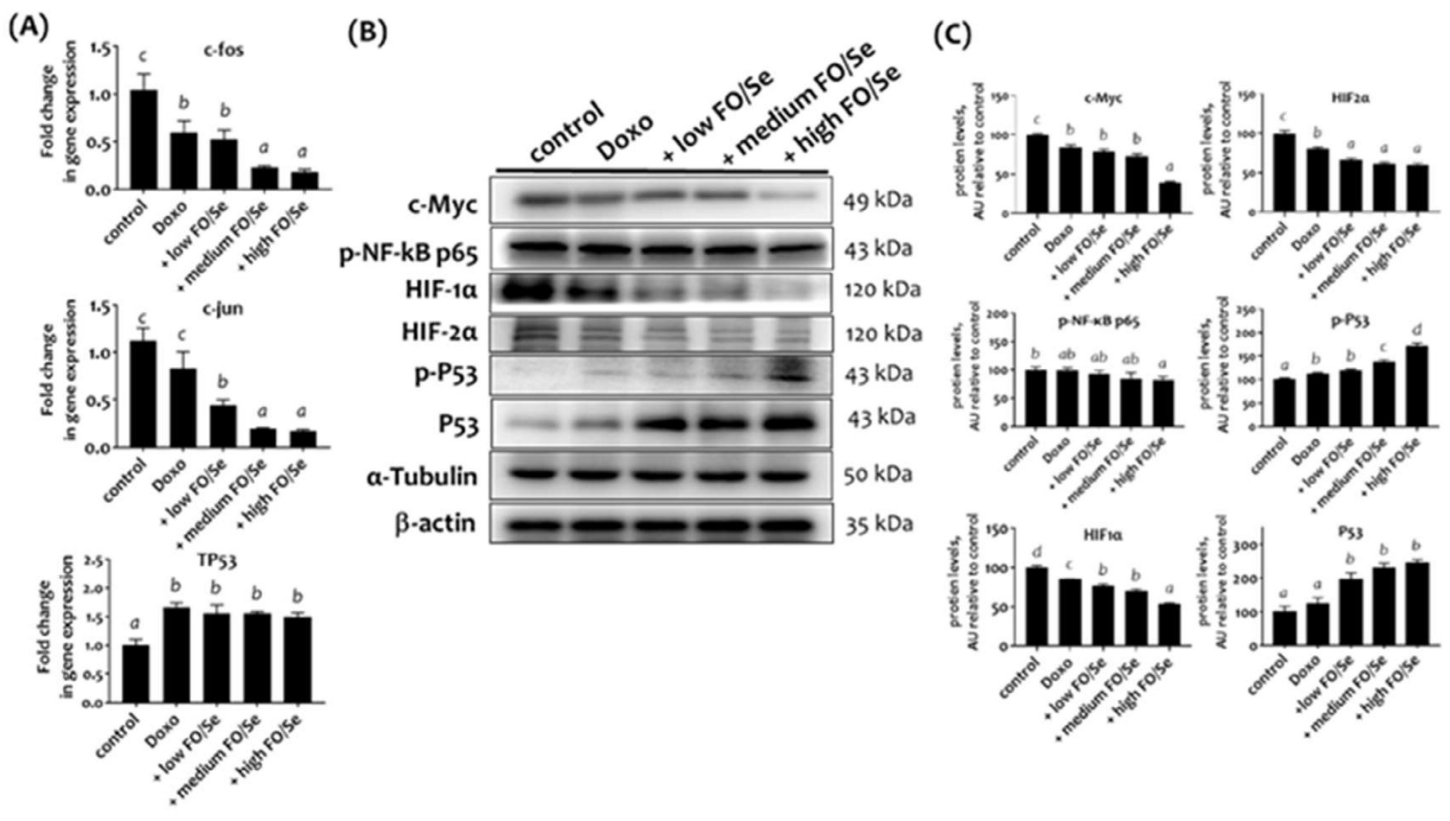
** (A) c-Jun, c-Fos, and TP53 mRNA levels in TNBC mouse tumor. (B) Expression of c-Myc, NF-κB p65, HIFs, and P53 proteins. (C) Densitometric analysis of three separate western blots.** Values are expressed as relative readings (mean ± standard error) from 3-4 mice in each group. Details of groups illustrated above are described in Figure 1. Superscript ^a, b, c, d^: Bars sharing the same superscript are not significantly different from each other; Bars with different superscript are significantly different from each other (*p* < 0.05).

Mice Tumors from mice in the doxorubicin groups expressed lower mRNA levels of nuclear transcription factors c-Jun and c-Fos than did those from control mice (Figure 4A). Combination treatment with doxorubicin and FO/Se resulted in significantly lower tumor mRNA expression than in mice treated with doxorubicin alone.

#### c-Myc, NF-κB p65, and HIFs proteins

Mice treated with doxorubicin expressed lower tumor levels of c-Myc compared to control mice. Mice treated with doxorubicin plus high-FO/Se had the lowest protein levels of c-Myc (Figure 4B). Mouse tumors in the doxorubicin group had non-significantly lower expression of p-NF-κB p65 protein compared to controls, while p-NF-κB p65 expression was significantly lower in mice treated with doxorubicin plus high-FO/Se.

Mice treated with doxorubicin had lower expression of tumor HIF-1α and HIF-2α than did controls (Figure 4B), and those treated with doxorubicin plus FO/Se expressed significantly lower levels of HIF-1α and HIF-2α protein than did those treated with doxorubicin alone.

### Combination treatment increased P53 tumor suppressor expression

TP53 mRNA levels were significantly higher in tumors from mice treated with doxorubicin alone or in combination with FO/Se than in control mice (Figure 4A). Mice treated with doxorubicin expressed higher levels of p-P53 protein than did those from control mice. Mice receiving combination treatment with doxorubicin and medium- or high-FO/Se expressed significantly higher tumor levels of p-P53 protein than did those treated with doxorubicin alone (Figure 4B).

### Combination treatment altered tumor immune checkpoints

**Figure 5:**
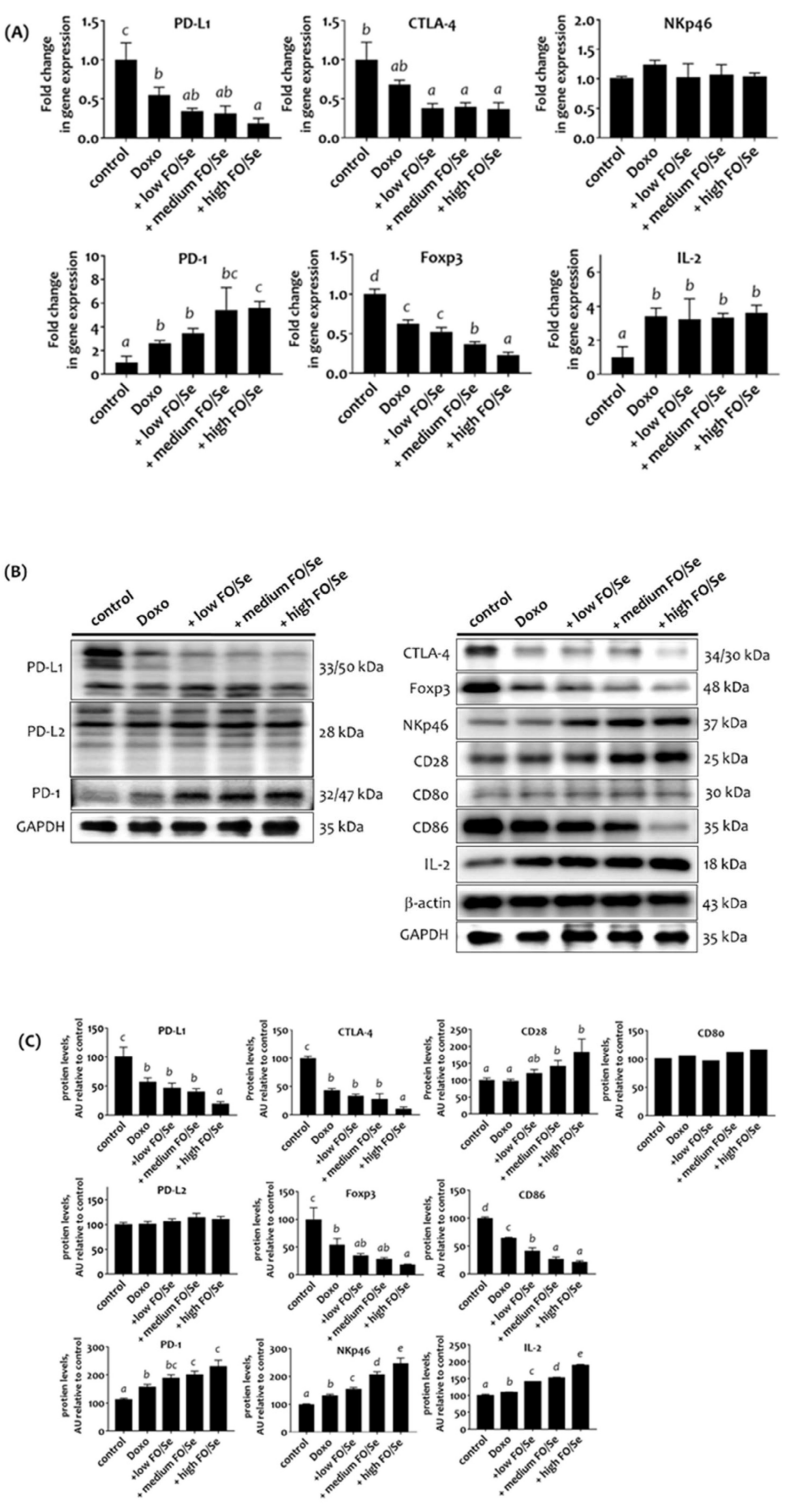
**(A) mRNA levels of PD-1/PD-L1, CTLA-4, Foxp3, and IL-2 in TNBC mouse tumor. (B) PD-1/PD-L1/PD-L2, and CTLA-4, Foxp3, Nkp46, CD28, CD80/86, and IL-2 protein levels. (C) Densitometric analysis of three separate western blots.** Values are expressed as relative readings (mean ± standard error) from 3-4 mice in each group. Details of groups illustrated are given in Figure 1. Superscript ^a, b, c, d^: Bars sharing the same superscript are not significantly different from each other; Bars with different superscript are significantly different from each other (*p* < 0.05).

Mice treated with doxorubicin expressed lower mRNA levels of PD-L1, CTLA-4, and Foxp3 than did control mice, while combined treatment with doxorubicin and FO/Se resulted in even lower mRNA levels (Figure 5A). No difference in natural killer-cells-activating receptor (NKp46) mRNA levels was observed between the 5 treatment groups. Tumors from mice treated with doxorubicin alone and in combination with FO/Se expressed higher mRNA levels of PD-1 and IL-2, and those treated with high- FO/Se had the highest PD-1 mRNA expression level.

Mice treated with doxorubicin alone or combination treatment had lower expression of tumor PD-L1, CTLA-4, Foxp3, and tumor-infiltrating dendritic cell (CD86) proteins than did controls, with the lowest expression in mice co-treated with high-FO/Se (Figures 5B, 5C). We observed a non-significant difference in tumor expression of PD-L2 and tumor-infiltrating dendritic cell (CD80) proteins between the 5 groups. Mice treated with doxorubicin had higher expression levels of PD-1, NKp46, and IL-2 proteins than did the control mice. Combination treatment at all concentrations of FO/Se resulted in higher PD-1, NKp46, CD28, and IL-2 expression than doxorubicin treatment alone.

### Combination treatment decreased cell proliferation and cell cycles, and cancer stemness

**Figure 6:**
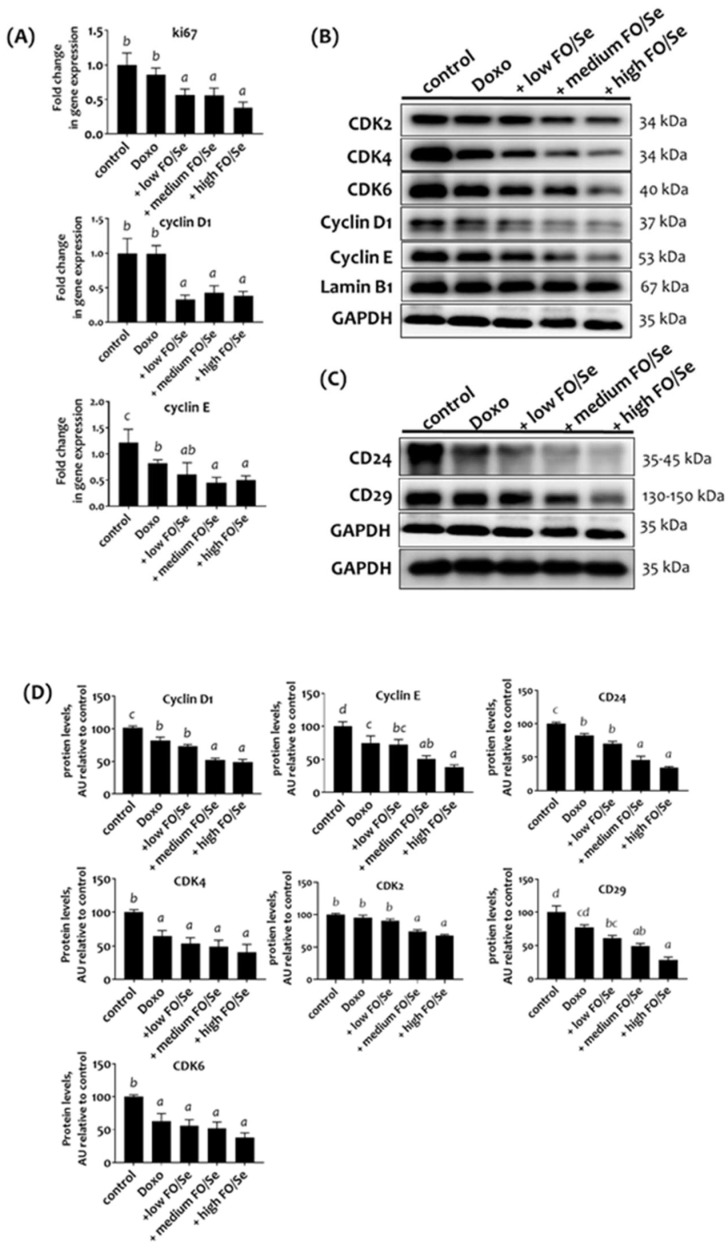
** (A) mRNA levels of Ki-67 and cyclin D1/E in TNBC mouse tumor. (B) Expression of CDK2/4/6 and cyclin D1/E proteins. (C) Expression of cancer stemness markers. (D) Densito-metric analysis of three separate western blots.** Values are expressed as relative readings (mean ± standard error) from 3-4 mice in each group. Details of groups illustrated are listed in Figure 1. Superscript ^a, b, c, d^: Bars sharing the same superscript are not significantly different from each other; Bars with different superscript are significantly different from each other (*p* < 0.05).

RT-qPCR analysis revealed a non-significantly lower Ki-67 mRNA level in tumors of doxorubicin-treated mice than in controls (Figure 6A). Mice treated with doxorubicin plus low, medium, or high doses of FO/Se expressed markedly lower levels of Ki-67 than those treated with doxorubicin alone.

We observed non-significant difference in tumor mRNA levels of cyclin D1 and lower levels of cyclin E in doxorubicin-treated mice than in controls (Figure 6A). Combination treatment with doxorubicin and FO/Se resulted in lower mRNA levels of cyclin D and E. Expression of the cell cycle proteins cyclin E was lower in mice treated with doxorubicin alone than in control mice and was markedly lower in those treated with doxorubicin plus medium- and high- FO/Se (Figure 6B). Lower expression of cyclin D1, cyclin-dependent kinase 4 (CDK4), and CDK6 was observed in mice treated with doxorubicin plus low-, medium-, and high-FO/Se compared to those treated with doxorubicin alone and controls.

The expression levels of cancer stem cell markers CD24 and CD29 were lower in doxorubicin-treated mice than in controls (Figure 6C), with lower expression in mice treated with doxorubicin plus FO/Se supplements than with doxorubicin alone.

## Discussion

**Figure 7:**
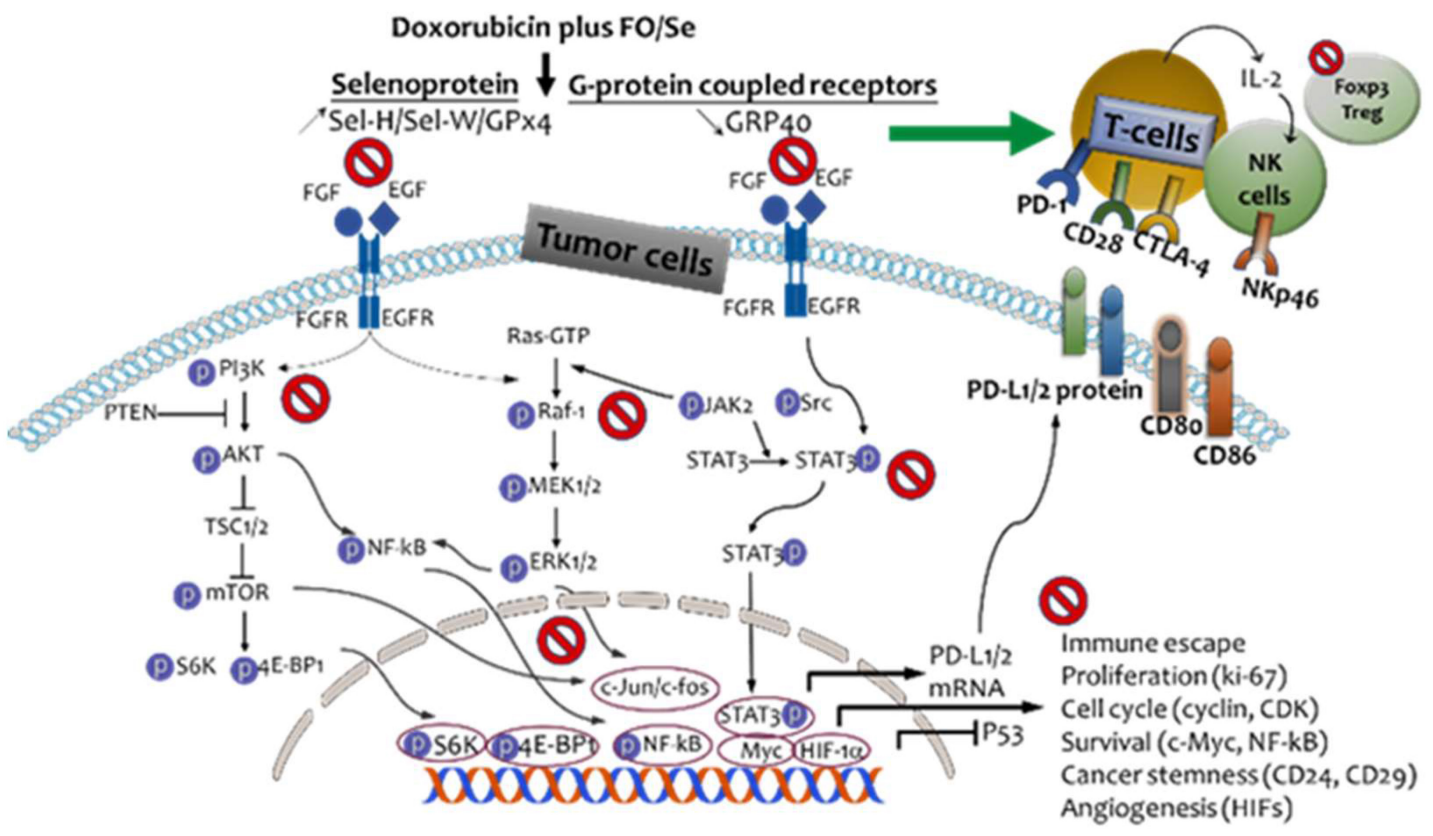
Schematic diagram showing combination treatment with FO and Se increases the therapeutic efficacy of doxorubicin against TNBC.

Evidence indicating that nutritional supplementation with FO and Se beneficially affects immune modulation and several anti-cancer-signaling cascades 12-15 suggests its potential usefulness for improving the effectiveness of doxorubicin in treating TNBC. In this investigation of combination treatment with doxorubicin and three doses of FO/Se in a mouse model, we observed that FO/Se increased the inhibitory effects of doxorubicin on the receptor tyrosine kinases EGFR and FGFR and increased its therapeutic targeting of downstream signaling pathways (PI3K/Akt/mTOR, Ras/MEK/ ERK, and JAK2/Src-1/STAT3) involved in tumor PD-1/PD-L1 immune regulation, cancer stemness, angiogenesis, and tumor growth, as shown in Figure 7.

Our results show that FO/Se treatment modulated the expression of multiple signaling molecules in a dose-dependent manner, although no significant difference was observed in tumor size between the 3 combination treatment groups. The sample size in this study may be too small to accurately reflect differences in tumor growth between these treatment groups. Further, TNBC tumor-bearing mice had significantly higher spleen and liver weights than did healthy controls and that combination treatment with FO/Se and anti-cancer agents exerted marked reductions in spleen and liver weights compared to anti-cancer agents alone; the results were consistent with that reported by previous study 14. Dramatic reductions in spleen, liver, and renal weights and alleviation of spleno-/hepatomegaly on combined treatment with FO/Se and doxorubicin may be attributed to reduction of leukemoid reaction, tumor immune cell infiltration, hypoxia-regulated HIF-1α induction, and tumor invasion in the present study. Following the combination treatment, the elevated levels of ALT and BUN were also reduced suggest FO/Se improved tumor or doxorubicin-induced adverse effects in liver and renal functions.

We observed that combination treatment with doxorubicin plus FO/Se resulted in higher tumor expression of Sel-H, Sel-W, and GPx4 than did doxorubicin alone. A recent study showed that Sel-H protein is a key regulator of cell cycle progression and prevents uncontrolled proliferation, suggesting an inhibitory effect of Sel-H in tumor progression 17. GPx4, a member of the Sel family, maintains redox homeostasis. GPx4 overexpression in hepatocellular carcinoma (HC) cells and an HC animal model has been shown to inhibit cell growth, angiogenesis, and expression of the tumor proliferation marker Ki-67 18,19. Tumor-bearing mice treated with Se have Se accumulation and higher pro-oxidative apoptosis in tumor tissues 20, and up-regulation of Sels expression in a dose-dependent manner may be partially responsible for the anti-TNBC effects of combination treatment.

Our previous study showed that combined treatment of FO/Se-bevacizumab results in the accumulation of EPA and DHA in tumor tissues and suppresses the expression of certain tumor G-protein-coupled chemokine receptors 15. Additionally, GPR-40 and GPR-120 are major G-protein coupled receptors for long-chain fatty acids and may have tumor-promoting activity 21. Overexpression of GPR-40 or GPR-120 is associated with the activation of PI3K/Akt and MAPK/ERK signaling, angiogenesis, and EMT, thus increasing chemoresistance in colorectal carcinoma and breast cancer cells 22,23. EPA was shown to suppress GPR-40 mRNA expression in MDA-MB-231 and MCF-7 human breast cancer cells 24. We observed here that of all the treatments tested, doxorubicin combined with high-FO/Se exerted the greatest inhibitory effect on GPR-40 mRNA expression compared with doxorubicin alone. However, this treatment did not affect the mRNA expression of GPR-120. Thus, targeting GPR-40 may provide an anti-cancer effect in TNBC tumors by doxorubicin-FO/Se combination treatment.

EGFR is a transmembrane receptor, and its overexpression has been observed in breast cancer tissues and cell lines, and EGFR silencing inhibits cancer cell proliferation 25. Aberrant FGFR activation was also observed in TNBC cell lines, and an FGFR inhibitor induces cell cycle arrest and apoptosis 26. Tumor Sel-H expression may be associated with the inhibitory effects of the EGFR and FGFR, and Sel-W negatively modulates EGFR expression in cell culture 18. DHA and EPA may be potential EGFR antagonists; they exert antiproliferative effects by inhibiting the EGFR pathway in cancer cells but not in normal cells 27,28. Induction of GPR-40 transactivated EGFR expression in human colorectal cancer tissues 29. Our previous study showed that mice treated with FO/Se plus bevacizumab have lower expression of EGFR and FGFR proteins in tumor tissues than mice treated with bevacizumab alone 14. The present study further shows that combination treatment modulates Sels and GPR-40 may lead to lower levels of phosphorylated EGFR and FGFR in tumors.

Furthermore, FGFR or EGFR can activate PI3K/Akt/mTOR and MAPK/ERK signaling. In-activation of tumor suppressors PTEN, TSC1, and TSC2 in TNBC is common and is associated with hyperactivation of PI3K/Akt/mTOR/4EBP1/p70S6K signaling pathway; phosphorylation of p70S6K/ 4EBP1 by mTOR has been shown to increase cancer cell survival and chemotherapy resistance in TNBC 30. In fact, PI3K suppression can down-regulate Akt but activate MAPK signaling; mTOR inhibition may up-regulate upstream EGFR/ FGFR and then reactivates Akt 31. Selenite, an inorganic form of Se, can inhibit the expression of tumor PI3K and phosphorylation of AKT in H22 tumor-bearing mice 32. Our results suggest that doxorubicin-FO/Se combination treatment leads to lower levels of PI3K, Akt, mTOR, p70S6K, and 4EBP1 phosphorylation along with higher expression of PTEN, TSC1, and TSC2 proteins. The observed dose-dependent activation or inhibition of these signaling molecules was likely due to the effects of FO/Se.

Activation of MAPK/ERK signaling cascade plays a vital role in transducing signals from cell-membrane receptors to the nucleus, thus associating with a poor TNBC prognosis 33. JAK2/Src-1/STAT3 signaling is also up-regulated by EGFR/FGFR activation that has been identified as an essential driver of TNBC proliferation, stemness, and chemoresistance. Unexpectedly, doxorubicin treatment was reported to clearly increase ERK and STAT3 phosphorylation levels *in vitro* 34,35. The results from earlier observations 15 and our current study show that compared to doxorubicin alone or low-dose FO/Se combination treatment, high-dose FO/Se treatment results in greater decreases in the expression of Ras, Raf, MEK1/2, and ERK1/2 proteins, as well as lower phosphorylation levels of JAK2, Src-1, and STAT3. These findings suggest that combination of FO and Se can enhances doxorubicin's anti-cancer effect is mediated by down-regulating MAPK/ERK and JAK2/Src-1/STAT3 signaling.

Furthermore, nuclear proto-oncogenes c-Fos and c-Jun are overexpressed in invasive breast cancer tumors compared to benign breast tissues 36. Aberrant expression of c-Myc together with other transcription factors is also commonly found in TNBC 37,38. The phosphorylation of p65 activates transcription factor NF-κB signaling; NF-κB is activated in breast cancer and interacts with other transcription factors such as STAT3, p53, and HIF-1α to modulate target genes 39. Se downregulates the activation of NF-κB and NF-κB-related target genes in prostate cancer cells 40, and increased Sel expression is associated with NF-κB inactivation 41. EPA is reported to downregulate NF-κB p65 signaling in ovarian tumors 42. A previous study showed that supplementation with inorganic Se decreased c-Myc expression in colon cancer cells 43. Our results show that combination treatment with doxorubicin-high dose FO/Se results in greatest downregulation of tumor c-fos, c-Jun, c-Myc, NF-κB p65, and HIFs. These findings suggest that FO/Se exerts a significant anti-TNBC effects partially through downregulating the oncoprotein transcription factors.

The cell proliferation-associated nuclear protein Ki-67 is also confirmed to be an independent prognostic factor in TNBC. Se compounds or Sels induce cancer stem cell apoptosis and reduce Ki-67 levels by modulating multiple signaling pathways 44-46. EPA and DHA suppress the self-renewal and growth of breast cancer stem cells 47. Our present findings indicate that combination doxorubicin-high dose FO/Se treatment results in greater inhibition of Ki-67, cyclin D1/CDK4/CDK6, cyclin E/CDK2, and cancer stem cells markers in tumor tissues, suggesting that FO/Se increases the effectiveness of doxorubicin treatment by arresting cancer growth and inhibiting cancer stemness.

Down-regulation of PD-L1 increases doxorubicin-induced apoptosis in TNBC cancer cells 48. T_reg_ expression of Foxp3+ within the tumor microenvironment is associated with worse overall survival and chemotherapeutic resistance in TNBC 49. T_reg_-mediated immunosuppressive activity is increased by the immune checkpoint molecule CTLA-4, natural killer (NK) cell inactivation, and increased consumption of IL-2 by T_regs_ and T_eff_ deprivation. Se increased the T-lymphocyte-mediated tumor cytotoxicity and NK cell activity to decreased PD-L1 levels 50. Our earlier study showed that combination treatment with anti-cancer agents and low-dose FO/Se resulted in a decrease in PD-L1 protein expression and increased PD-1 protein expression in tumors. The increases in PD-1 expression with combination treatment may involve in the activation of T-cells and NK-cells in tumor tissues 14. Meanwhile, FO/Se treatment resulted in higher levels of plasma IFN-γ and IL-2 together with lower IL-6 in doxorubicin-treated tumor-bearing mice than does doxorubicin treatment alone. Our present study further demonstrates that FO/Se supplementation inhibits the expression of PD-L1, CTLA-4, Foxp3, and CD86 in tumors. Additionally, FO/Se increase the expression levels of PD-1, NKp46, CD28, and IL-2.

## Conclusions

The findings of this preclinical study significantly expand our understanding of the regulation of tumor microenvironment and immune checkpoint molecules upon doxorubicin-FO/Se combination therapy in murine TNBC tumors. We observed that supplemental FO/Se increases the anti-tumor response of doxorubicin through multiple mechanisms: 1) inducting Sels expression and inhibiting GPR-40 mRNA; 2) suppressing receptor tyrosine kinase EGFR and FGFR; 3) decreasing PI3K/Akt/mTOR/p70S6K/4EBP1, Raf/MEK/ERK1/2, and JAK/c-Src/STAT3 signaling; 4) activating tumor suppressor proteins PTEN, TSC1/2, and P53; 5) inhibiting nuclear oncoprotein transcriptional factors; and 6) regulating PD-1/PD-L1/CTLA-4 signaling. Therefore, chemotherapeutic doxorubicin-FO/Se combination treatment should be considered a potential therapeutic strategy in doxorubicin-treated patients with TNBC. Due to TNBC heterogeneity, immune cell infiltration into tumors should be characterized using flow cytometry and immunohistochemistry staining in future studies.

## Supplementary Materials

The following data are available online at www.mdpi.com/xxx/s1: Table S1: List of the primer sequences used for qRT-PCR analysis of specific genes; Table S2: List of antibodies used in this study; Figure S1: Comparison of plasma ALT and BUN in all groups. Figure S2: supplementary set of figures showing all original western blots.
