# Supplementary material for: Fish Oil and Selenium with Doxorubicin Modulates Expression of Fatty Acid Receptors and Selenoproteins, and Targets Multiple Anti-Cancer Signaling in Triple-negative Breast Cancer Tumors
Source: Int J Med Sci. 2022 Nov 14;19(14):2044–57. doi: 10.7150/ijms.75848 (PMC9724242; doi:10.7150/ijms.75848)
Supplement: Supplementary file 1 — The following data are available online at www.mdpi.com/xxx/s1: Table S1: List of the primer sequences used for qRT-PCR analysis of specific genes; Table S2: List of antibodies used in this study; Figure S1: Comparison of plasma ALT and BUN in all groups. Figure S2: supplementary set of figures showing all original western blots. [file ijmsv19p2044s1.pdf]

Supplementary Materials and Methods

Table S1. List of the primer sequences used for qRT-PCR analysis of specific genes

Gene name	primer sequences (5′→3′)	
	Forward	Reverse
Sel-H	GCTGCGCTCGGACACA	TCGTGGAGGGCCCTTTTA
GPx4	CTCCATGCACGAATTCTAG	ACGTCAGTTTTGCCTCATG
GRP40	GCCTCGGATGGCTACAAGT	CCTTCGCTCTCTATGTACTGCC
GRP120	ACCGCATAGGAGAAATCTCATGG	GATCTGGTGGCTCTCAGAGTA
c-fos	AGTCAAGGCCTGGTCTGTGT	ATCAGCTGCACTAGATACAATCC
c-jun	TACAGTCTCTATTGCAGTTTGTAAC	ACTGCATGGACCTAACATTCG
N-cadherin	GTTCTCCACTTGATTGCCATTGA	GATCGAGAGCTGATAGCCCG
Vimentin	TGGAGTCACTTCCTCTGGTTG	CAAGGTCATCGTGATGCTGAG
PD-1	TTCAGGTTTACCACAAGCTGG	TGACAATAGGAAACCGGGAA
PD-L1	GAAGTCCAGCTCCTCATA	TTCTGCTCAACAAGTATGTC
PD-L2	CTGCACGTCTCCATCATA	GGTATGAACATCGGCTCA
CTLA-4	CCGAGTCTGTGTGGGTTCAA	GAGCAGAGTAAAACCCAACAGGAT
FoxP3	TACGAGTGGATGGTGCGCTG	AGGTTGTGCCGGATGGAGTTC
IL-2	TGTGTTGTAAGCAGGAGGTACA	GATGGATAGCCTTCTGTCAAAGC
Ki-67	CTGCCTGCGAAGAGAGCATC	AGCTCCACTTCGCCTTTTGG
TP53	CAGGTTATCTTGTGTCAGT	GAGGCAGGTCAATATCAG
GAPDH	GTATGACTCCACTCACGGCAA	CTTCCCATTCTCGGCCTTG
E-cadherin	#MP2 00650, Sino Biological, Wayne, NJ, USA	

Table S2. List of antibodies used in this study

Sel-H (1:1000, orb317826, biorbyt)<br>Sel-W (1:1000, GTX48717, GeneTex)<br>EGFR (1:1000, #2193153, Millipore)<br>p-Tyr1068-EGFR (1:1000, #2234, Cell signaling)<br>FGFR (1:1000, #9740, Cell signaling)<br>p-FGFR (1:1000, GTX32182, GeneTex)<br>GAPDH (1:2000, MAB 374, Millipore)<br>β-actin (1:4000, MAB 1501, Millipore)<br>p-Tyr199/458-PI3K (1:1000, #4228, Cell signaling),<br>p-Ser473-Akt (1:1000, #4058, Cell signaling)<br>p-Thr308-Akt (1:1000, #9275, Cell signaling)<br>mTOR (1:1000, GTX101557, GeneTex)<br>p-Ser2448-mTOR (1:1000, #5536, Cell signaling)<br>PTEN (1:1000, #9552, Cell signaling)<br>p-Ser385-PTEN (1:1000, #9551, Cell signaling)	p-Tyr1007/1008-Jak2 (1:500, #3776, Cell signaling)<br>STAT3 (1:2000, #4904, Cell signaling)<br>p-Tyr705-STAT3 (1:1000, #9131, Cell signaling)<br>p-NF-◎B p65 (1:1000, #3033, Cell signaling)<br>c-Myc (1:1000, sc-40, Santa Cruz)<br>HIF-1α (1:500, ab42091, Abcam)<br>HIF-2α (1:1000, ab109616, Abcam)<br>α-Tubulin (1:2000, ab4074, Abcam)<br>Cyclin D1 (1:1000, sc-450, Santa Cruz Bio-technology)<br>Cyclin E (1:1000, sc-247, Santa Cruz Bio-technology)<br>CDK-2 (1:1500, sc-6248, Santa Cruz Bio-technology)<br>CDK-4,6 (1:1500, sc-23896, Santa Cruz Bio-technology)<br>CDK-6 (1:1500, sc-7961, Santa Cruz Bio-technology)

TSC1 (1:1000, #4906, Cell signaling)	Lamin B1 (1:2000, E-AB-31901, Elab-science)
TSC2 (1:1000, #4308, Cell signaling)	CD24 (1:2000, #2514189, Millipore)
4E-BP-1 (1:1000, #9644, Cell signaling)	CD29 (1:2000, ab179471, Abcam)
p-Thr37/46-4E-BP1 (1:1500, #2855, Cell signaling)	PD-1 (1:1000, 18106-1-AP, Proteintech)
p70S6K (1:1000, #9202, Cell signaling)	PD-L1 (1:1000, 66248-1-lg, Proteintech)
p-Thr421-Ser424-p70S6K (1:1000, #9204, Cell signaling)	PD-L2 (1:1000, 18251-1-AP, proteintech)
	CD80 (1:1000, ab215166, Abcam)
Ras (1:1000, 3233-S, Epitmics)	CD28 (1:1000, ab205136, Abcam)
p-Ser338-c-Raf1(1:1000, #9427, Cell signaling)	CD86 (1:1000, ab112490, Abcam)
p-Ser217/221-MEK1/2 (1:1000, #9154, Cell signaling)	Foxp3 (1:1000, ab450,Abcam)
	IL-2 (1:1000, 60306-1-lg, Proteintech)
ERK1/2 (1:1000, GTX59618, GeneTex)	P53 (1:1000, ab26, Abcam)
p-Thr202-Tyr204-ERK (1:1000, #GTX59568, GeneTex)	p-P53 (1:1000, #2525, Cell signaling)
p-Tyr416-c-Src (1:1000, #2101, Cell signaling)	

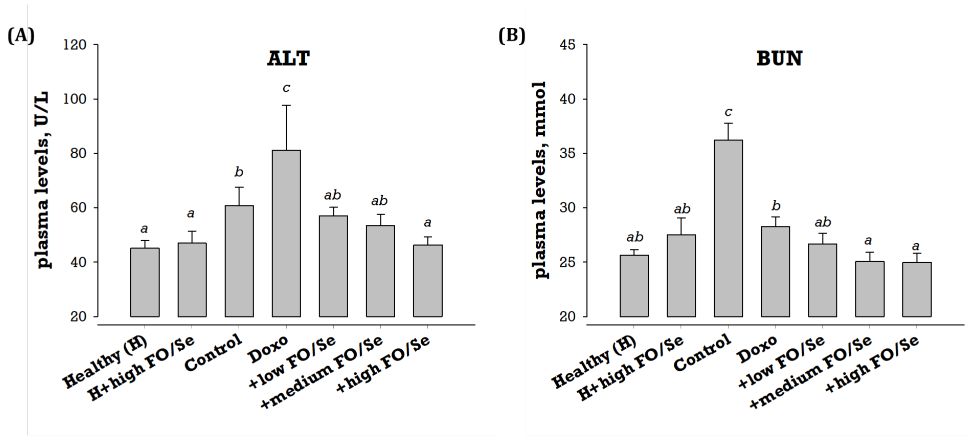

Figure S1. Comparison of plasma ALT and BUN in all groups.
Healthy non-tumor–bearing mice were assigned as Healthy (H) and H+high FO/Se groups and treated with or without high dose-FO/Se by oral gavage. Additionally, tumor-bearing mice were allocated to the following groups: control group, saline injected; Doxo group, injected intra-peritoneally with doxorubicin; +low FO/Se, +medium FO/Se, and +high FO/Se groups were injected intraperitoneally with doxorubicin together with a low, medium, or high dose of FO/Se p.o. Superscript [a, b, c, d]: Bars sharing the same superscript are not significantly different from each other; Bars with different superscript are significantly different from each other ($p < 0.05$). Doxo, doxorubicin; FO, fish oil; Se, selenium.

Figure S2. Supplementary set of figures showing all original western blots.
Tumor-bearing mice were randomized into 5 weight-matched groups as follows: 1) Control group, saline injected; 2) Doxo group, injected intraperitoneally with doxorubicin; 3) +low FO/Se, +medium FO/Se, and +high FO/Se groups were injected intraperitoneally with doxorubicin together with a low, medium, or high dose of fish oil/selenium. Doxo, doxorubicin; FO, fish oil; Se, selenium. Western blotting of protein expression in tumor tissues (n = 3 mice per group) and each band in blotting membrane represents an individual mouse.

Figure S2. Supplementary set of figures with all original western blots

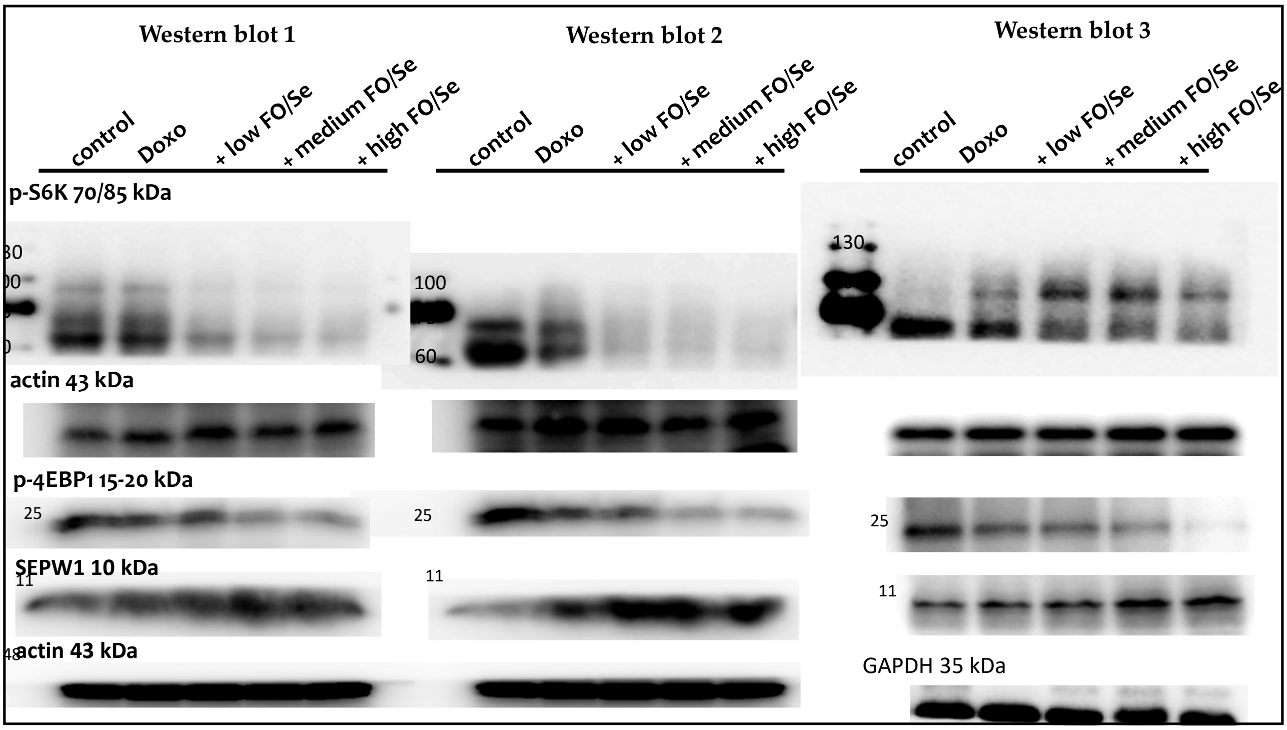

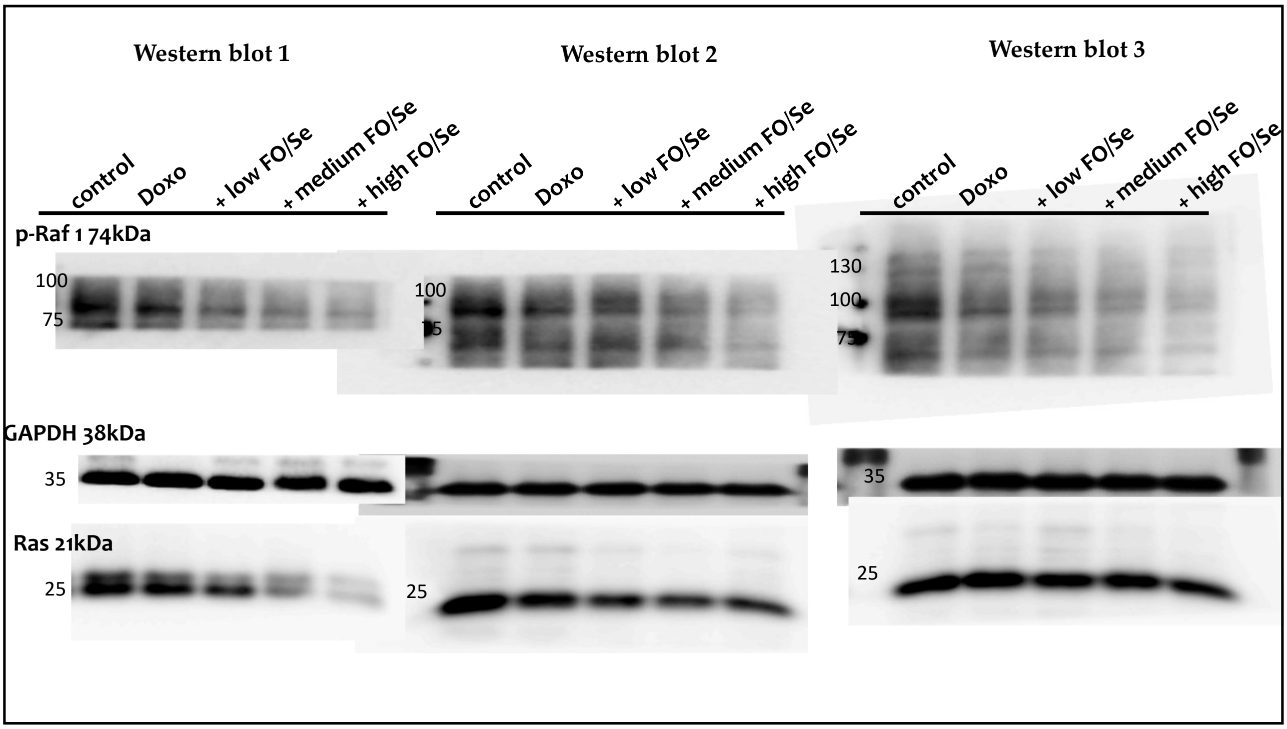
Western blot 1
Western blot 2
Western blot 3
control
Doxo
+ low FO/Se
+ medium FO/Se
+ high FO/Se
p-Raf 1 74kDa
100
75
130
GAPDH 38kDa
35
Ras 21kDa
25

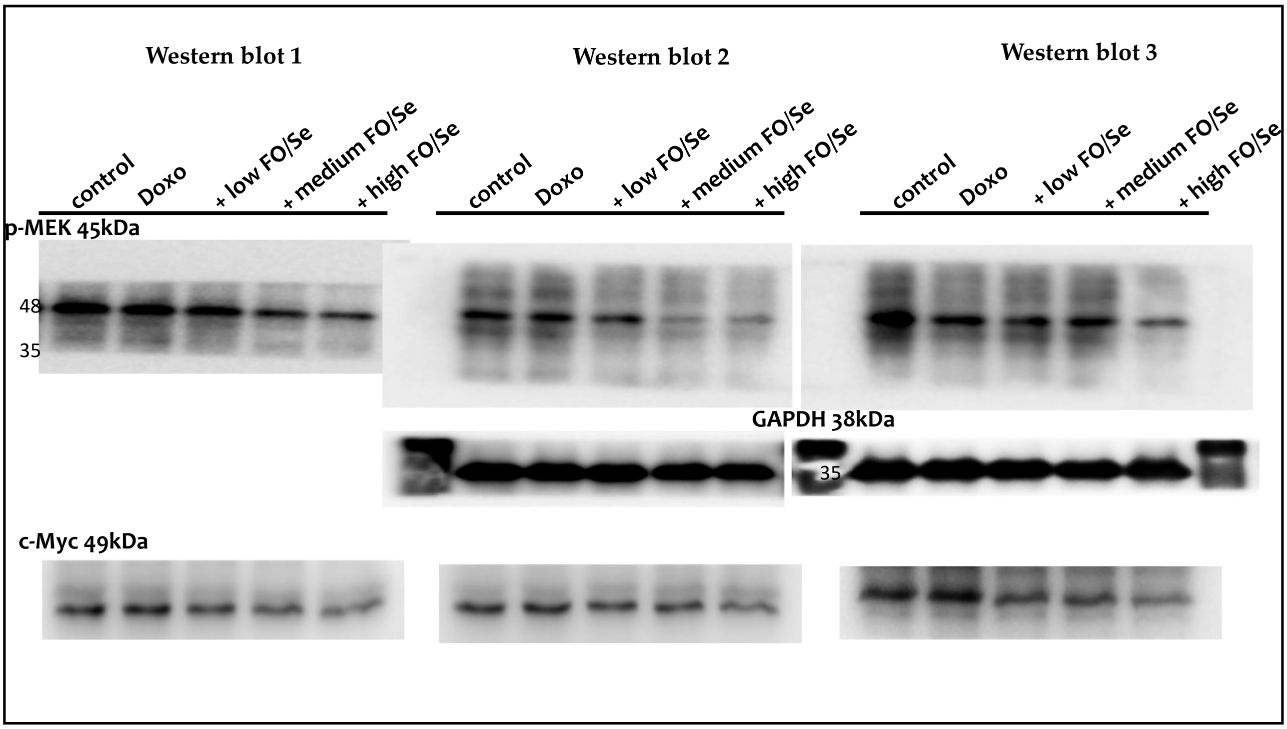
Western blot 1
Western blot 2
Western blot 3
control
Doxo
+ low FO/Se
+ medium FO/Se
+ high FO/Se
p-MEK 45kDa
48
35
GAPDH 38kDa
c-Myc 49kDa

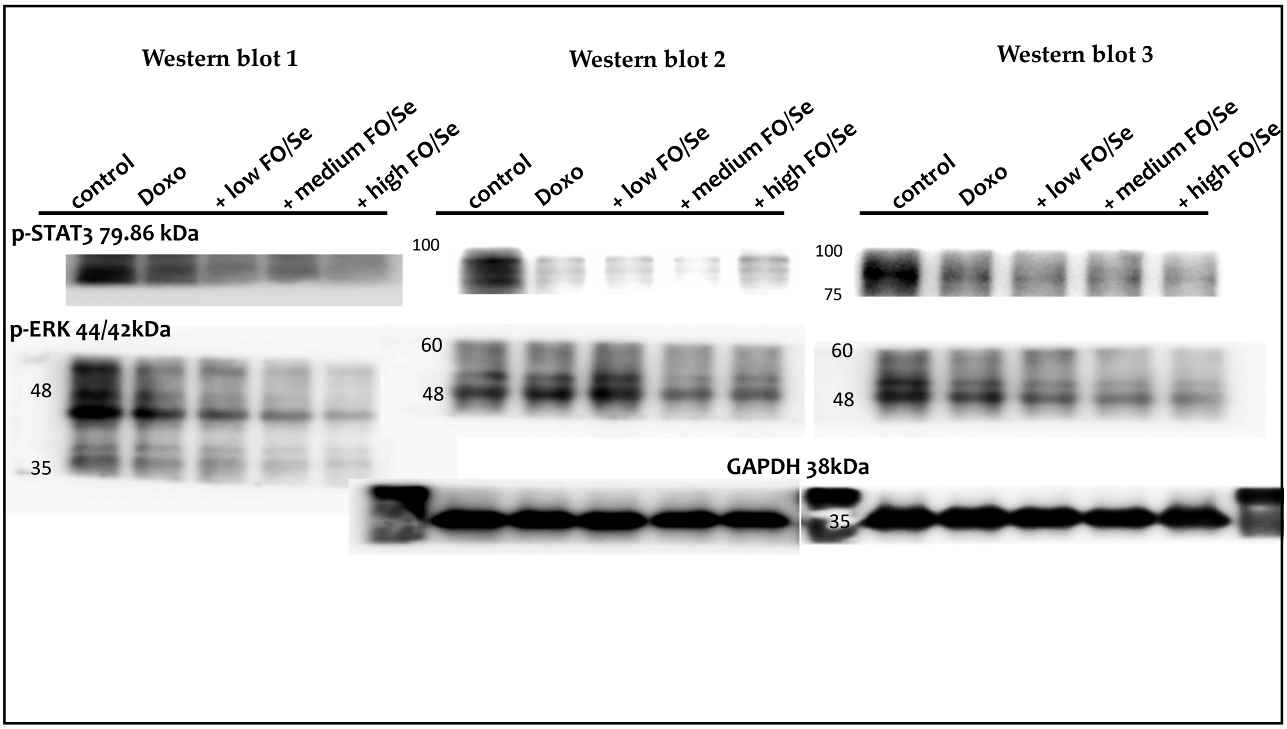
Western blot 1
Western blot 2
Western blot 3
control
Doxo
+ low FO/Se
+ medium FO/Se
+ high FO/Se
p-STAT3 79.86 kDa
p-ERK 44/42kDa
GAPDH 38kDa
100
75
60
48
35

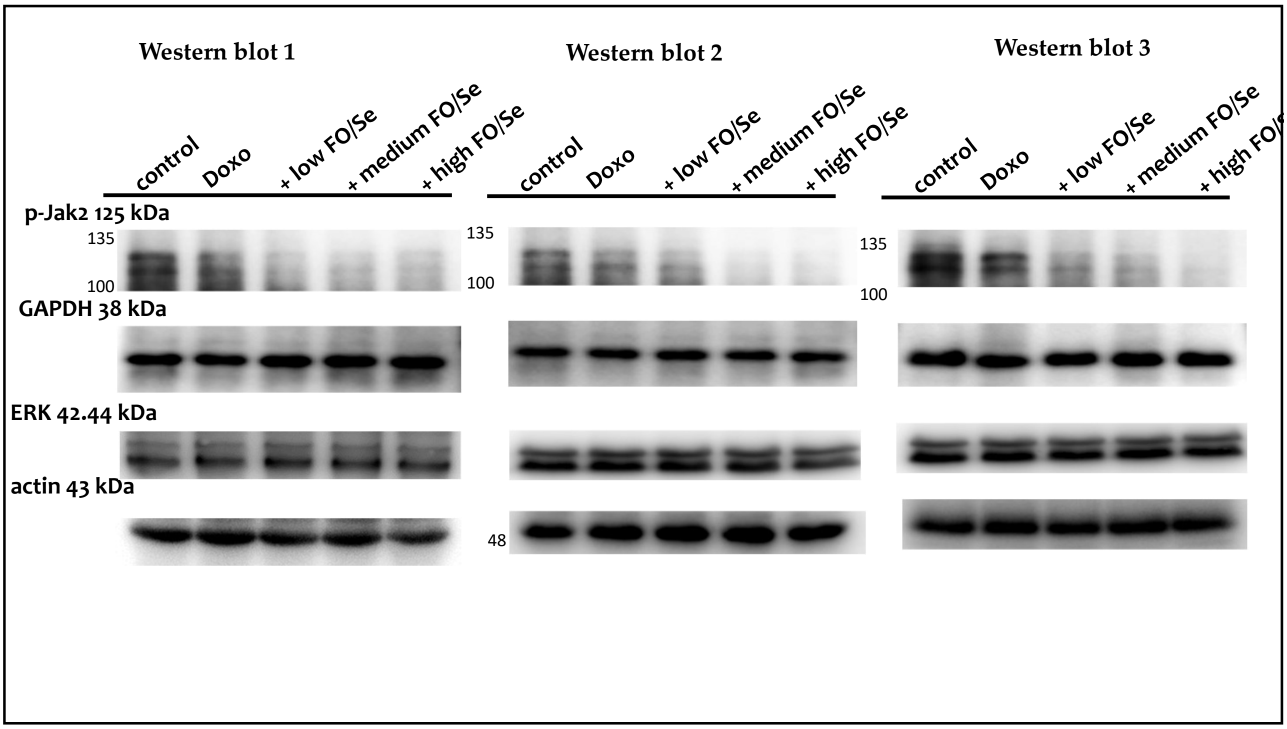
Western blot 1
Western blot 2
Western blot 3
control
Doxo
+ low FO/Se
+ medium FO/Se
+ high FO/Se
p-Jak2 125 kDa
GAPDH 38 kDa
ERK 42.44 kDa
actin 43 kDa
135
100
48

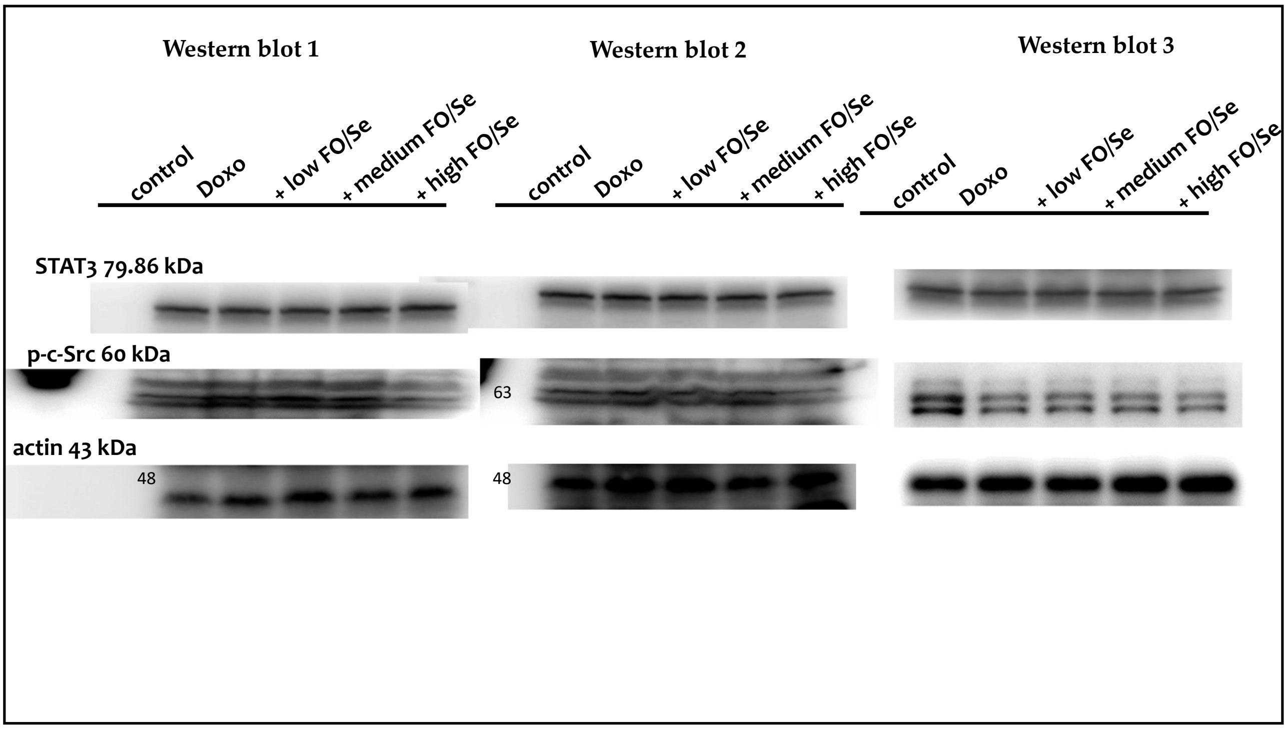
Western blot 1
Western blot 2
Western blot 3
control
Doxo
+ low FO/Se
+ medium FO/Se
+ high FO/Se
STAT3 79.86 kDa
p-c-Src 60 kDa
63
actin 43 kDa
48

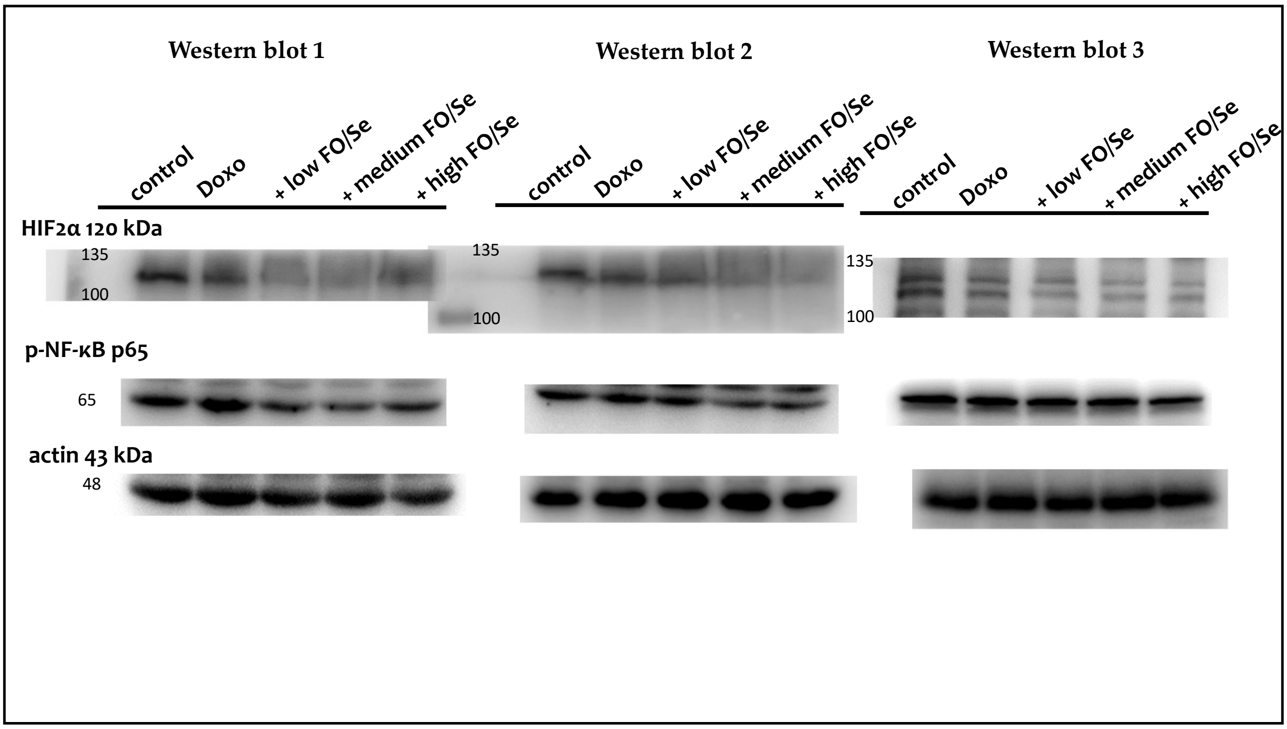
Western blot 1
Western blot 2
Western blot 3
control
Doxo
+ low FO/Se
+ medium FO/Se
+ high FO/Se
HIF2α 120 kDa
135
100
p-NF-κB p65
65
actin 43 kDa
48

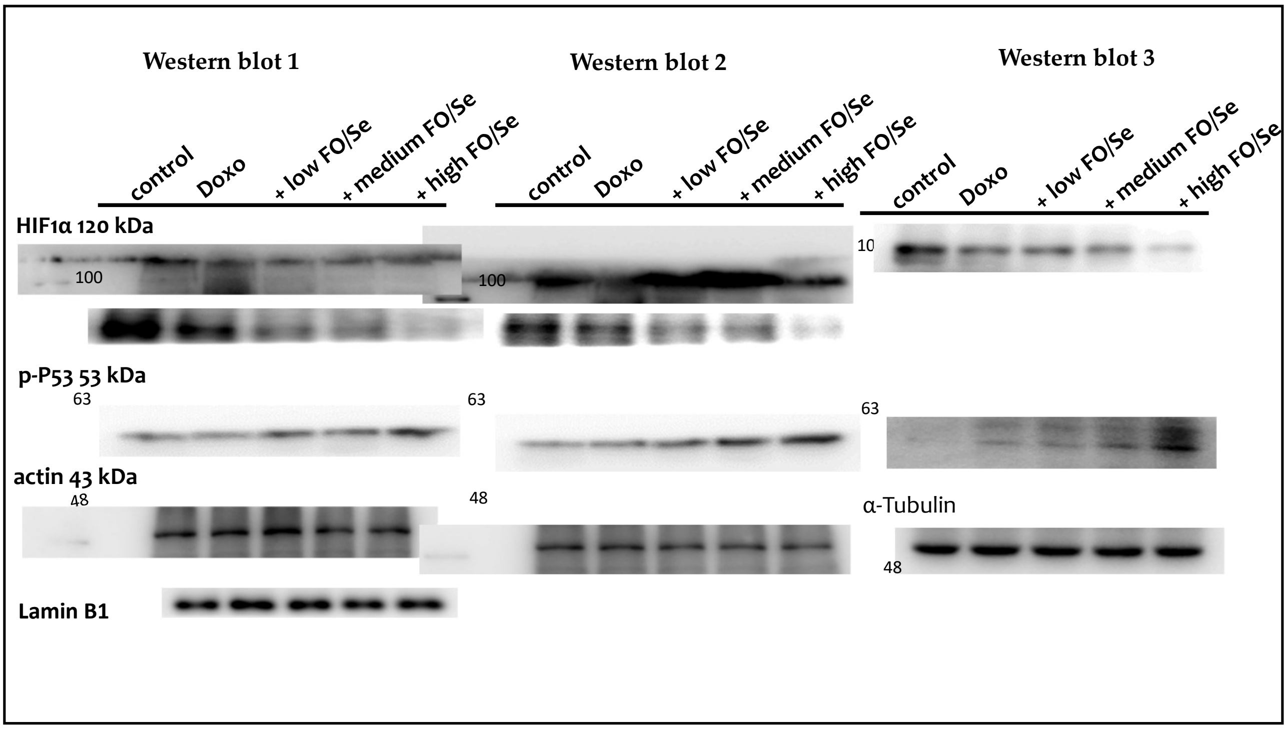

Western blot 1
Western blot 2
Western blot 3
control
Doxo
+ low FO/Se
+ medium FO/Se
+ high FO/Se
HIF1α 120 kDa
100
10
p-P53 53 kDa
63
actin 43 kDa
48
α-Tubulin
Lamin B1

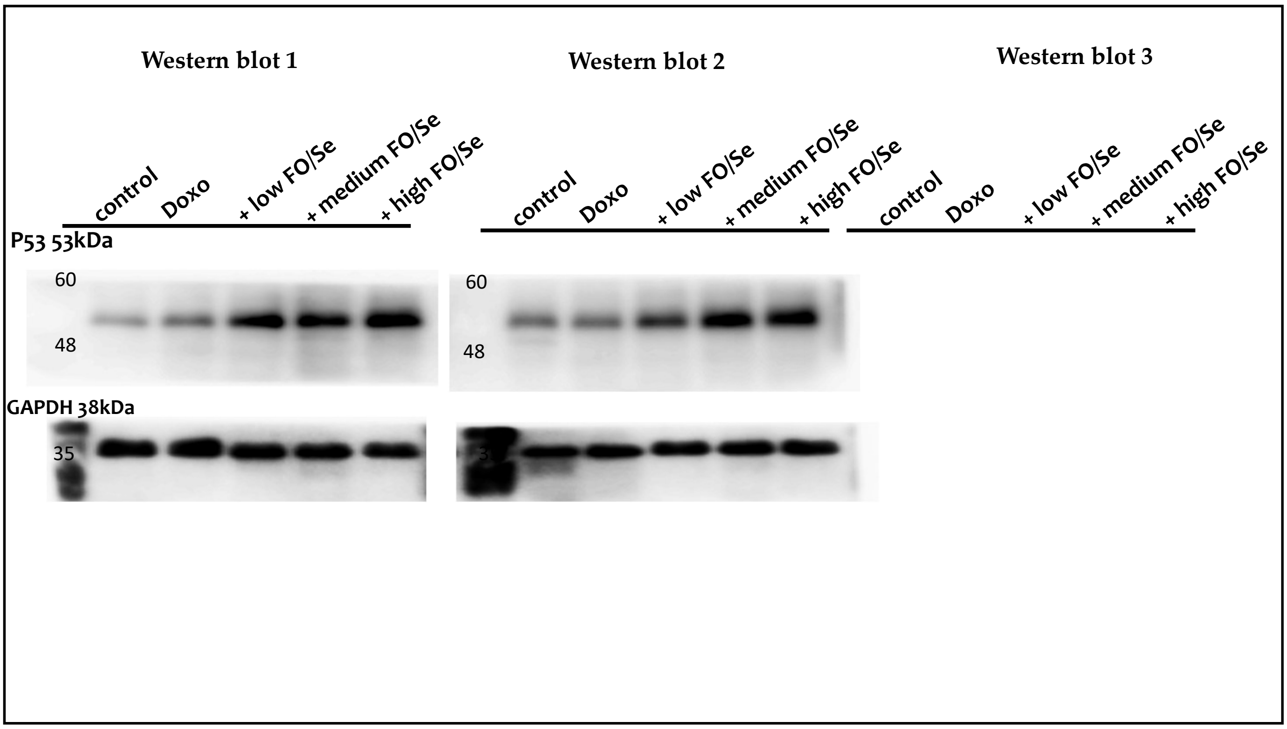

Western blot 1
Western blot 2
Western blot 3
control
Doxo
+ low FO/Se
+ medium FO/Se
+ high FO/Se
P53 53kDa
60
48
GAPDH 38kDa
35

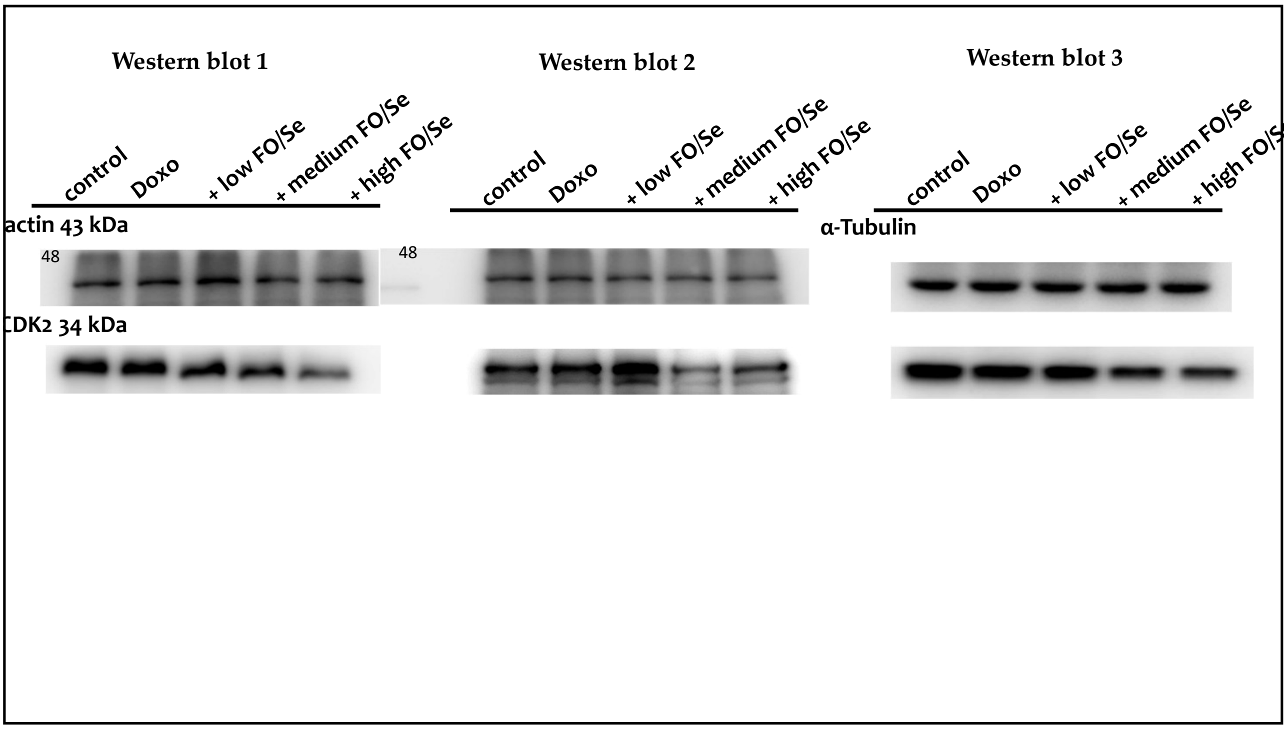
Western blot 1
Western blot 2
Western blot 3
control
Doxo
+ low FO/Se
+ medium FO/Se
+ high FO/Se
actin 43 kDa
48
CDK2 34 kDa
α-Tubulin

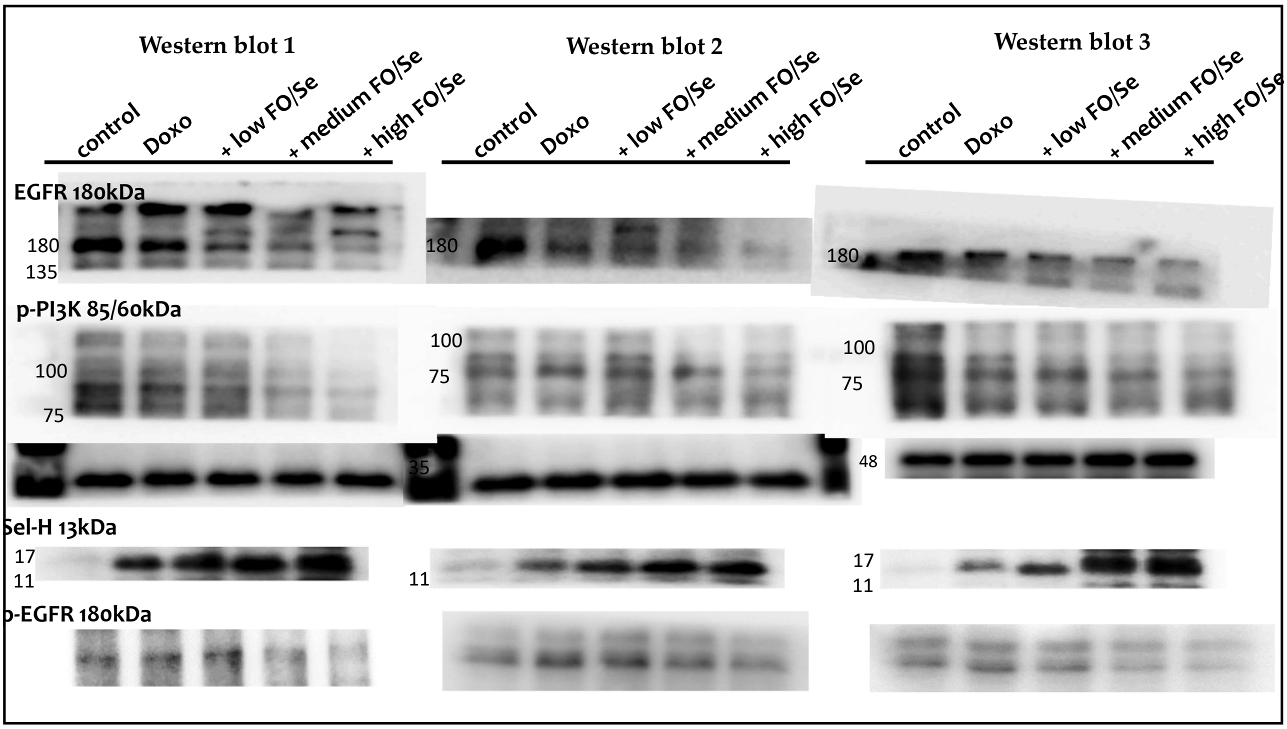
Western blot 1
Western blot 2
Western blot 3
control
Doxo
+ low FO/Se
+ medium FO/Se
+ high FO/Se
EGFR 180kDa
180
135
p-PI3K 85/60kDa
100
75
35
48
Sel-H 13kDa
17
11
p-EGFR 180kDa

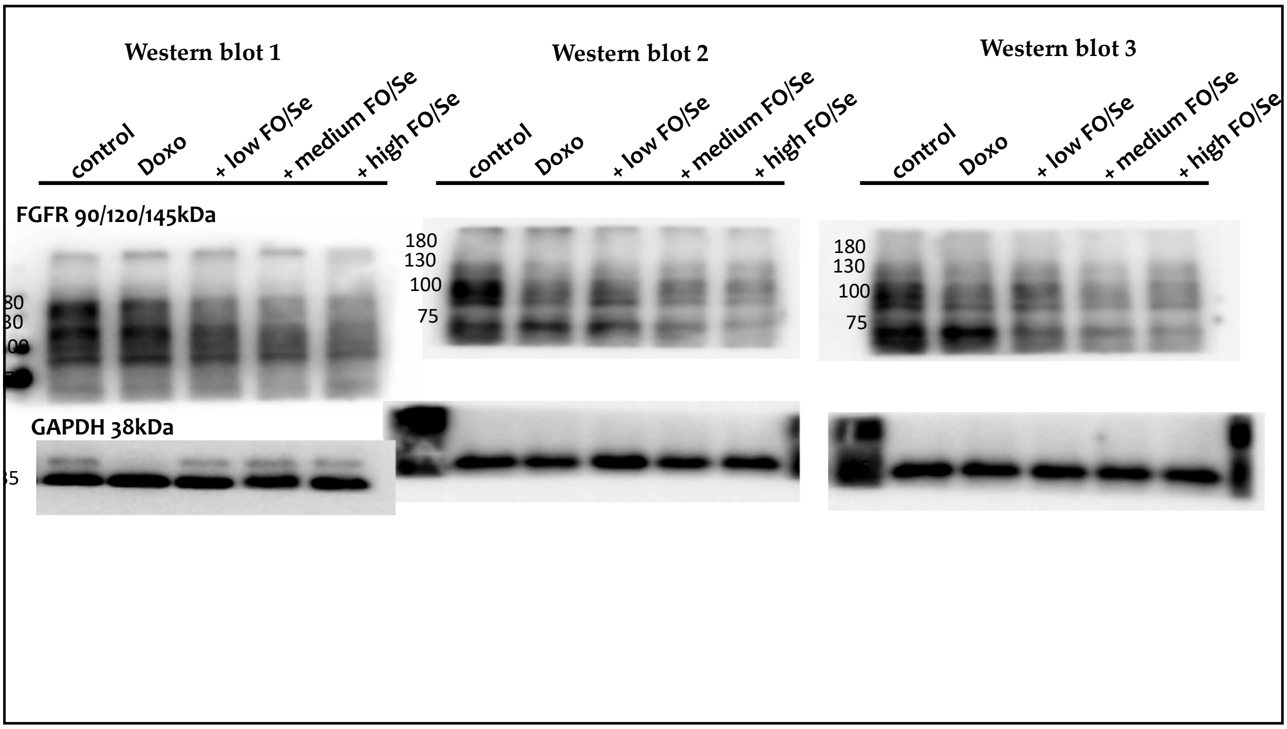
Western blot 1
Western blot 2
Western blot 3
control
Doxo
+ low FO/Se
+ medium FO/Se
+ high FO/Se
FGFR 90/120/145kDa
180
130
100
75
GAPDH 38kDa

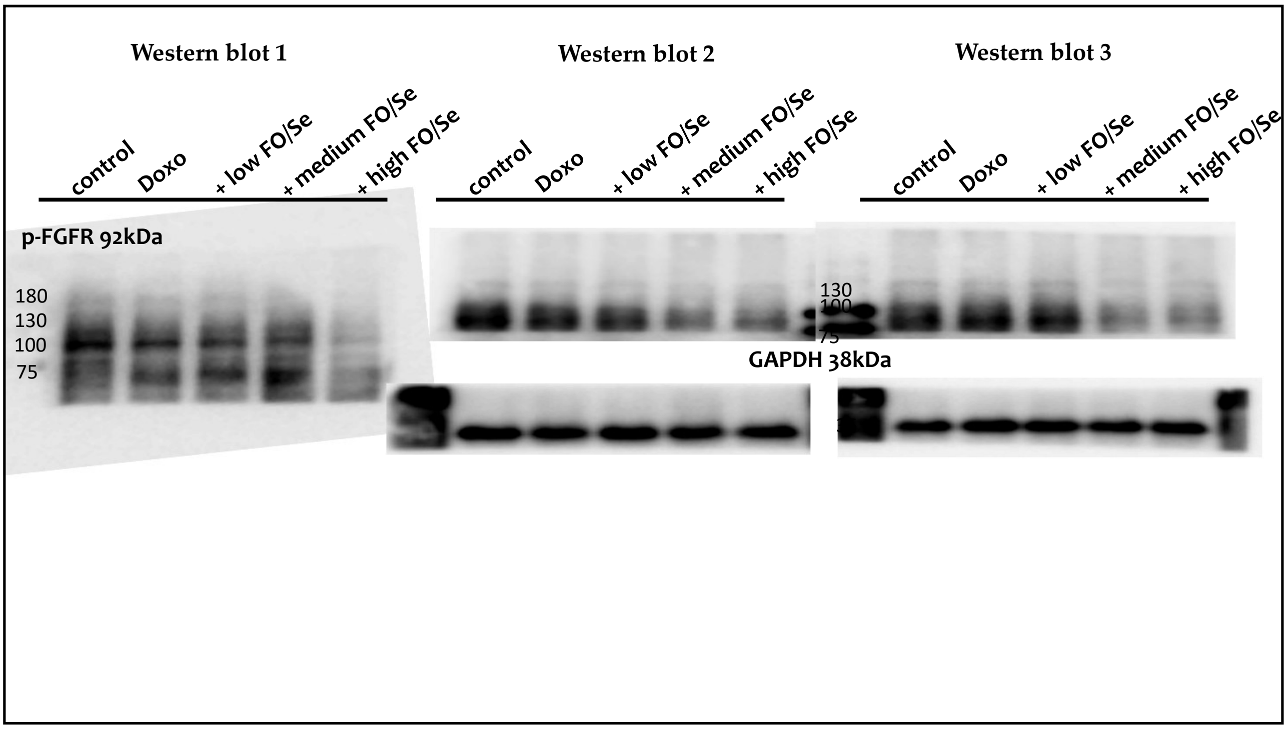
Western blot 1
Western blot 2
Western blot 3
control
Doxo
+ low FO/Se
+ medium FO/Se
+ high FO/Se
p-FGFR 92kDa
180
130
100
75
GAPDH 38kDa

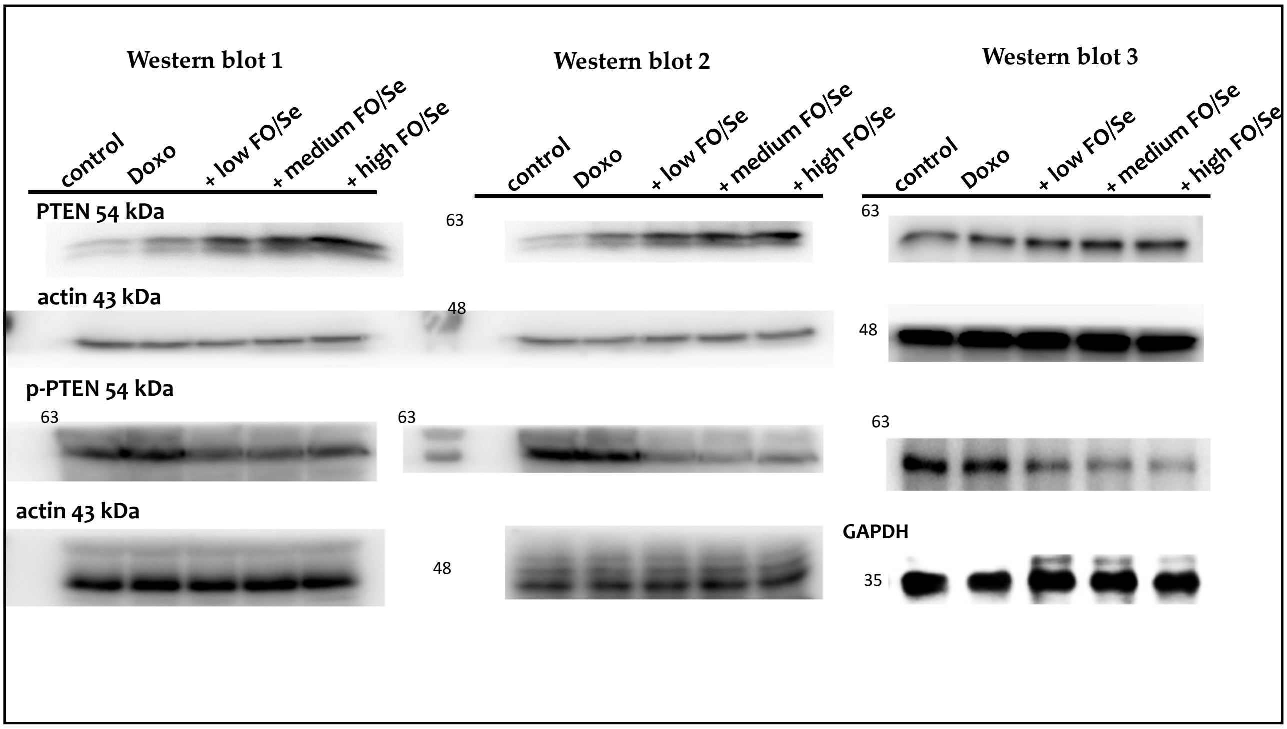
Western blot 1
Western blot 2
Western blot 3
control
Doxo
+ low FO/Se
+ medium FO/Se
+ high FO/Se
PTEN 54 kDa
actin 43 kDa
p-PTEN 54 kDa
actin 43 kDa
GAPDH
63
48
35

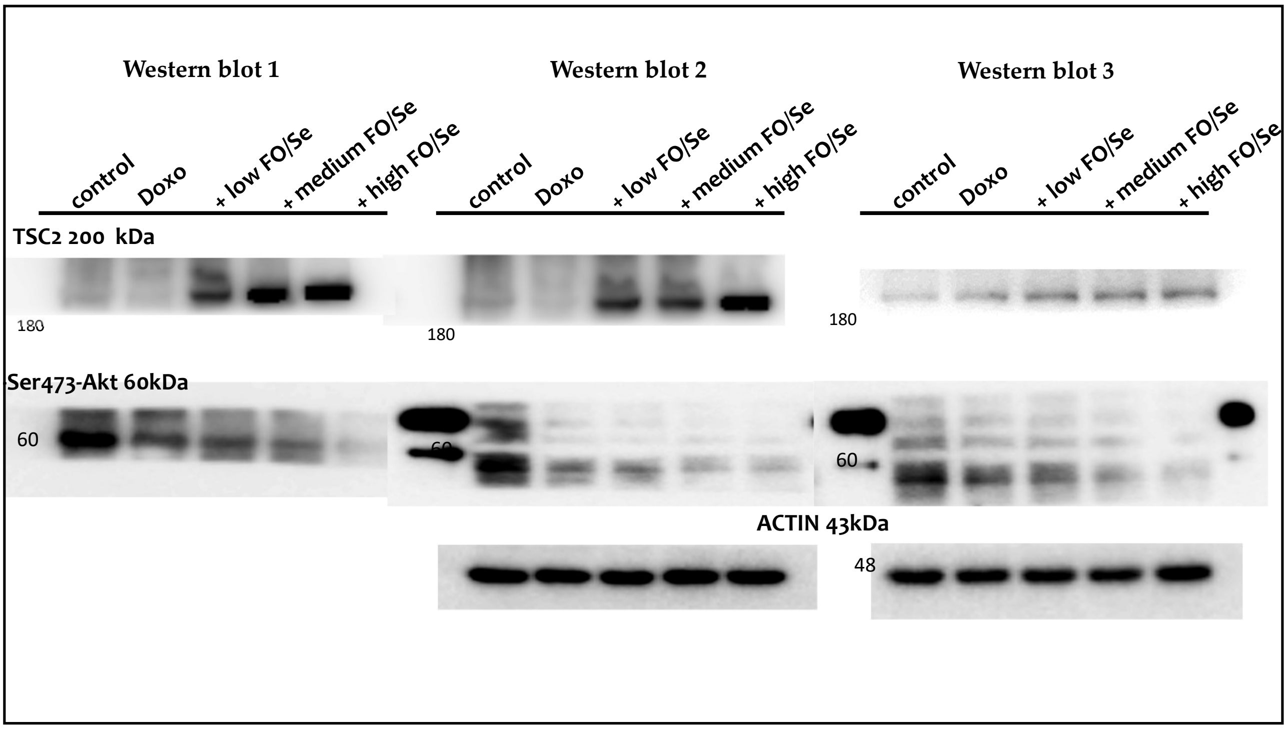
Western blot 1
Western blot 2
Western blot 3
control
Doxo
+ low FO/Se
+ medium FO/Se
+ high FO/Se
TSC2 200 kDa
Ser473-Akt 60kDa
ACTIN 43kDa
180
60
48

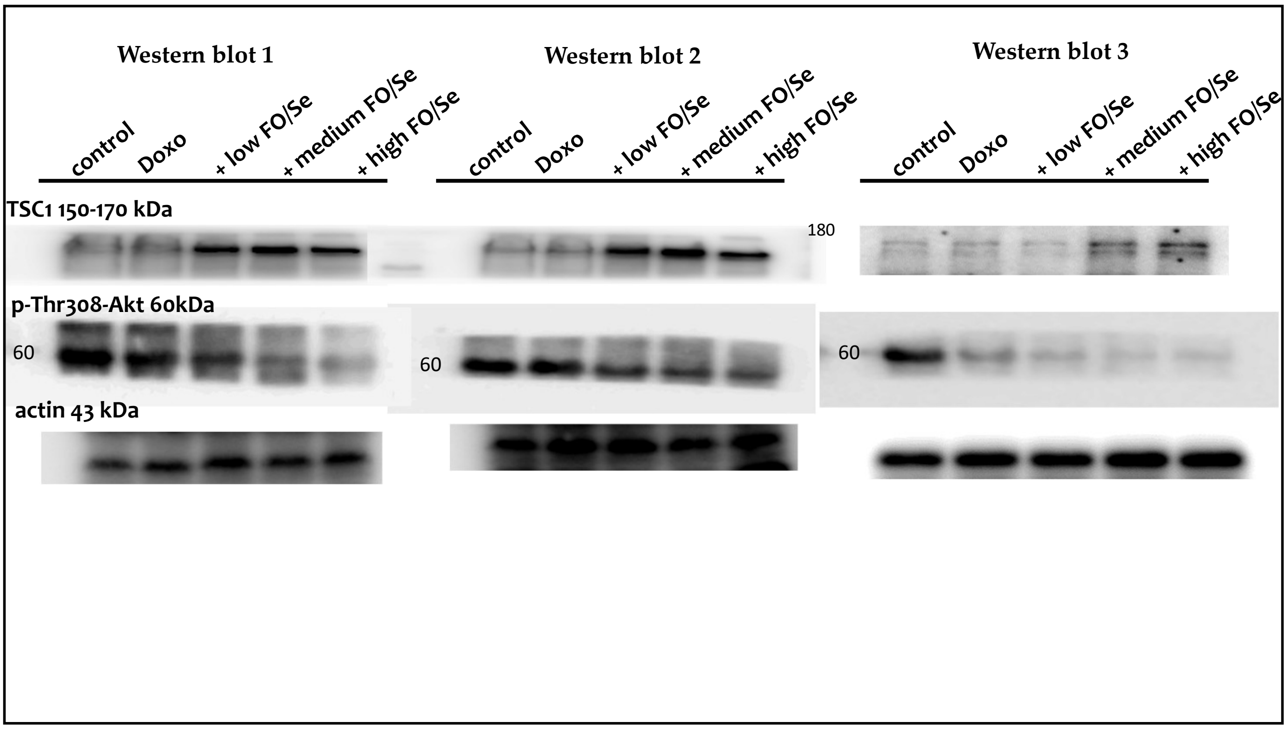
Western blot 1
Western blot 2
Western blot 3
control
Doxo
+ low FO/Se
+ medium FO/Se
+ high FO/Se
TSC1 150-170 kDa
180
p-Thr308-Akt 60kDa
60
actin 43 kDa

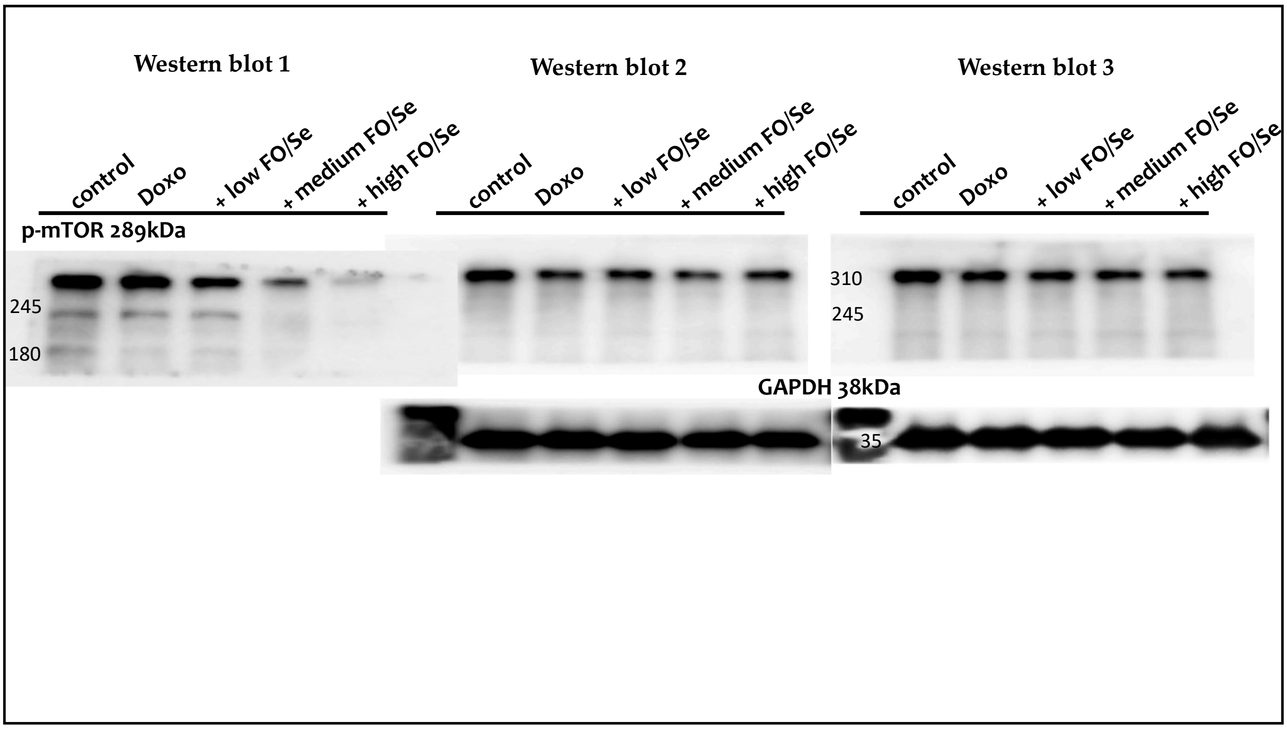
Western blot 1
Western blot 2
Western blot 3
control
Doxo
+ low FO/Se
+ medium FO/Se
+ high FO/Se
p-mTOR 289kDa
245
180
310
GAPDH 38kDa
35

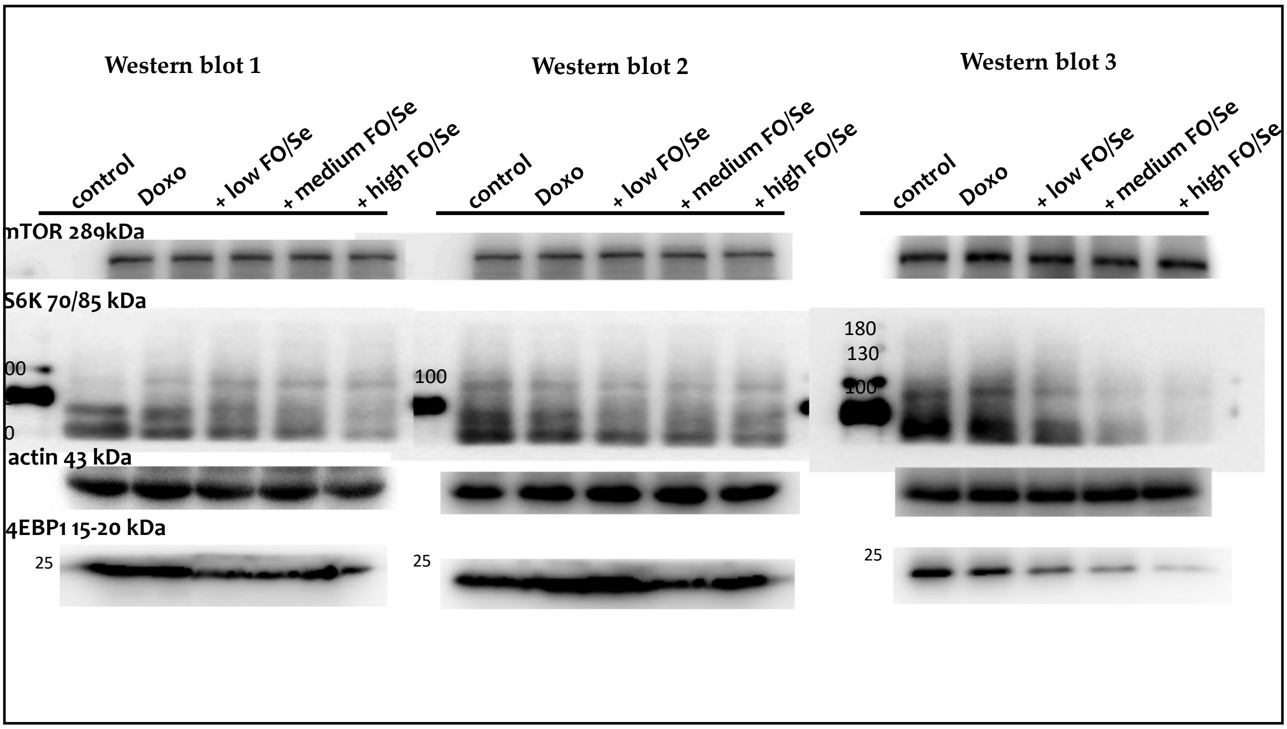
Western blot 1
Western blot 2
Western blot 3
control
Doxo
+ low FO/Se
+ medium FO/Se
+ high FO/Se
mTOR 289kDa
S6K 70/85 kDa
actin 43 kDa
4EBP1 15-20 kDa
180
130
100
25

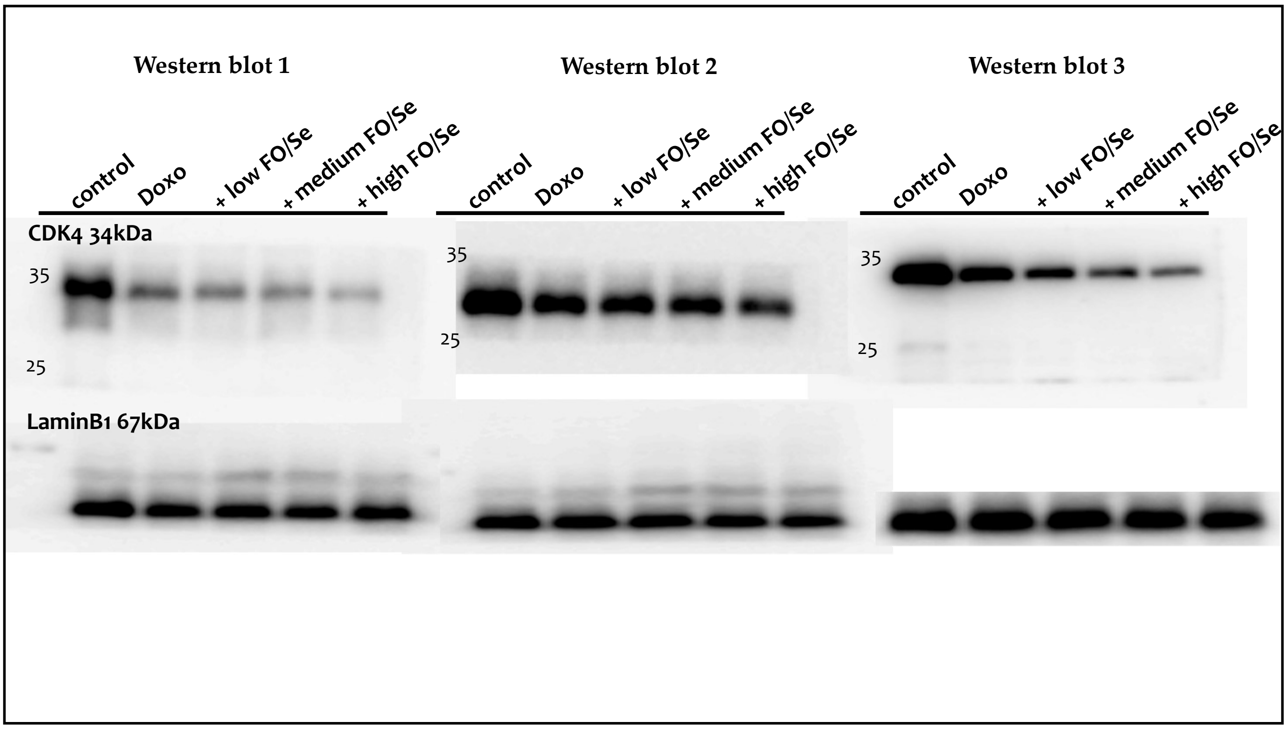
Western blot 1
Western blot 2
Western blot 3
control
Doxo
+ low FO/Se
+ medium FO/Se
+ high FO/Se
CDK4 34kDa
LaminB1 67kDa
35
25

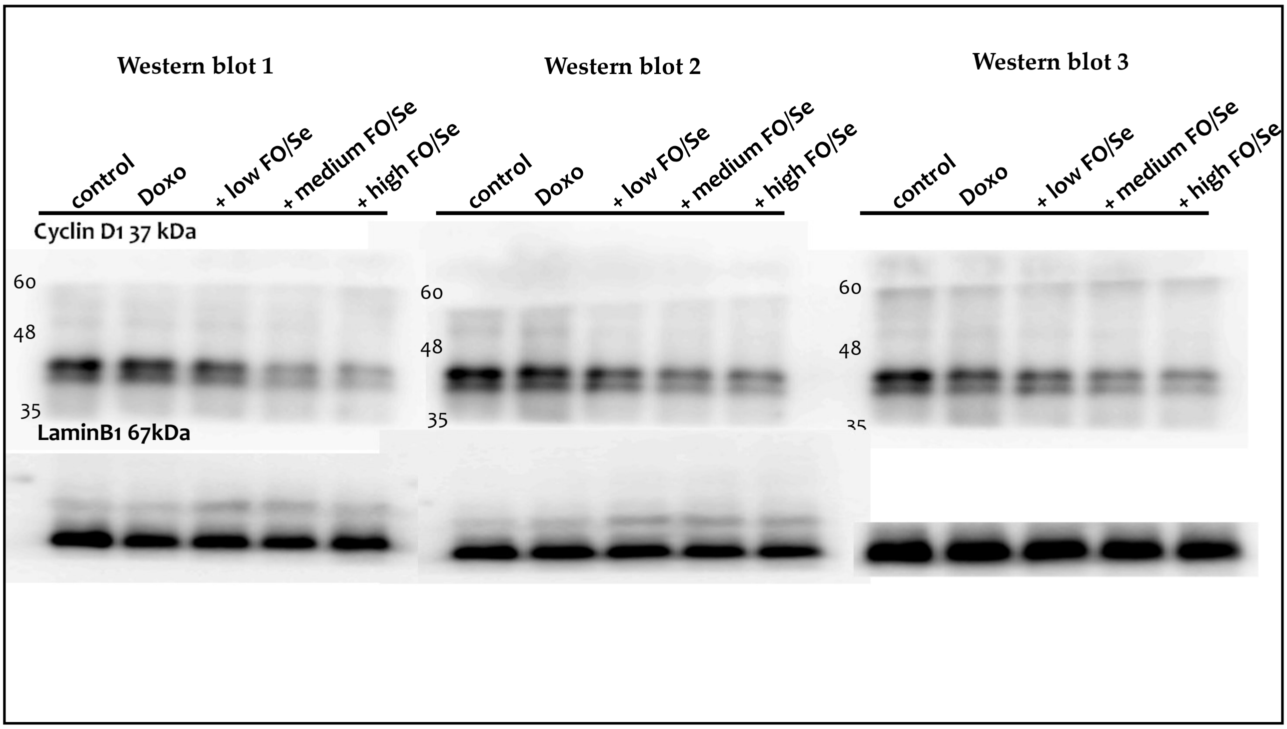
Western blot 1
Western blot 2
Western blot 3
control
Doxo
+ low FO/Se
+ medium FO/Se
+ high FO/Se
Cyclin D1 37 kDa
60
48
35
LaminB1 67kDa

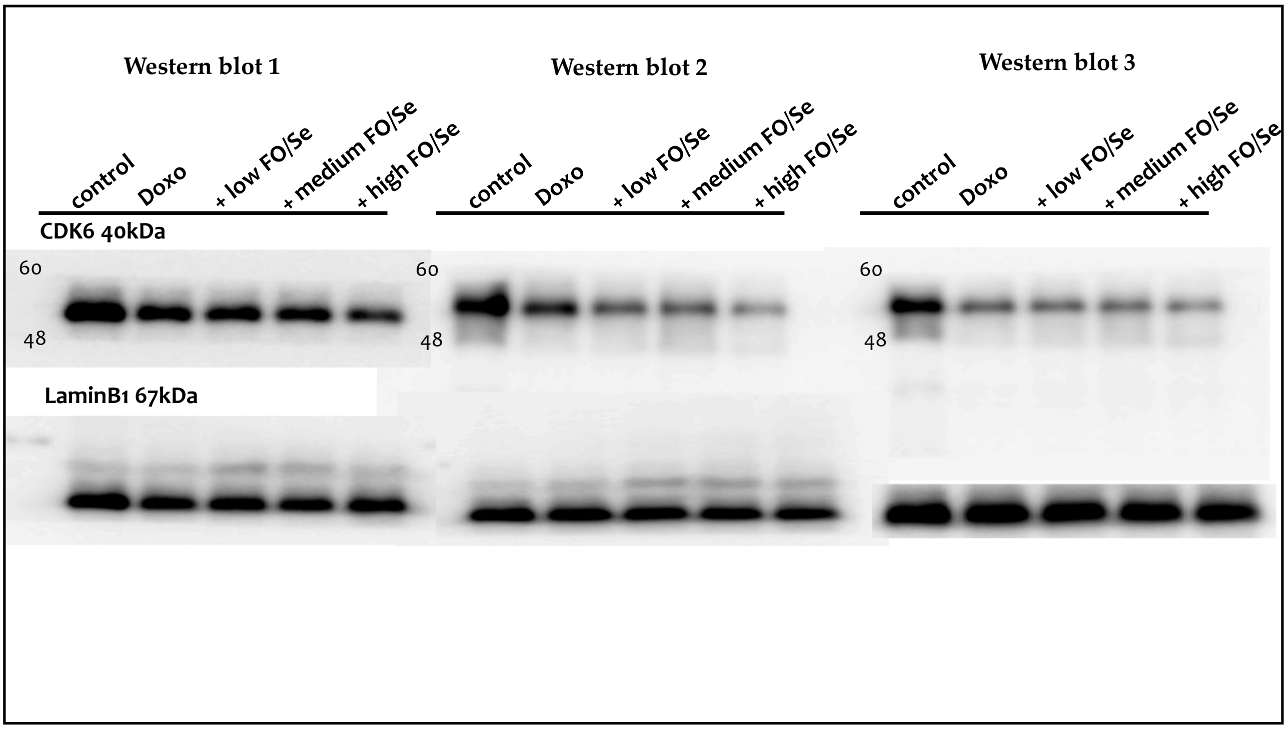
Western blot 1
Western blot 2
Western blot 3
control
Doxo
+ low FO/Se
+ medium FO/Se
+ high FO/Se
CDK6 40kDa
60
48
LaminB1 67kDa

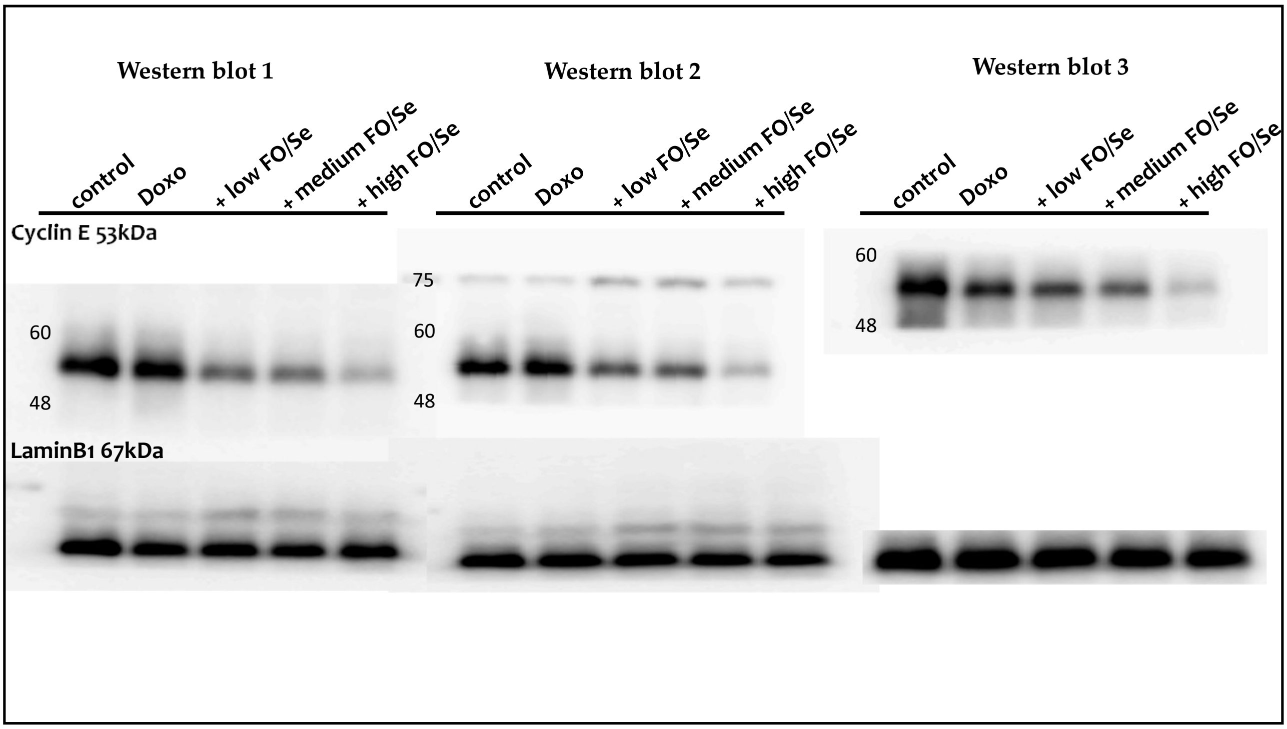
Western blot 1
Western blot 2
Western blot 3
control
Doxo
+ low FO/Se
+ medium FO/Se
+ high FO/Se
Cyclin E 53kDa
75
60
48
LaminB1 67kDa

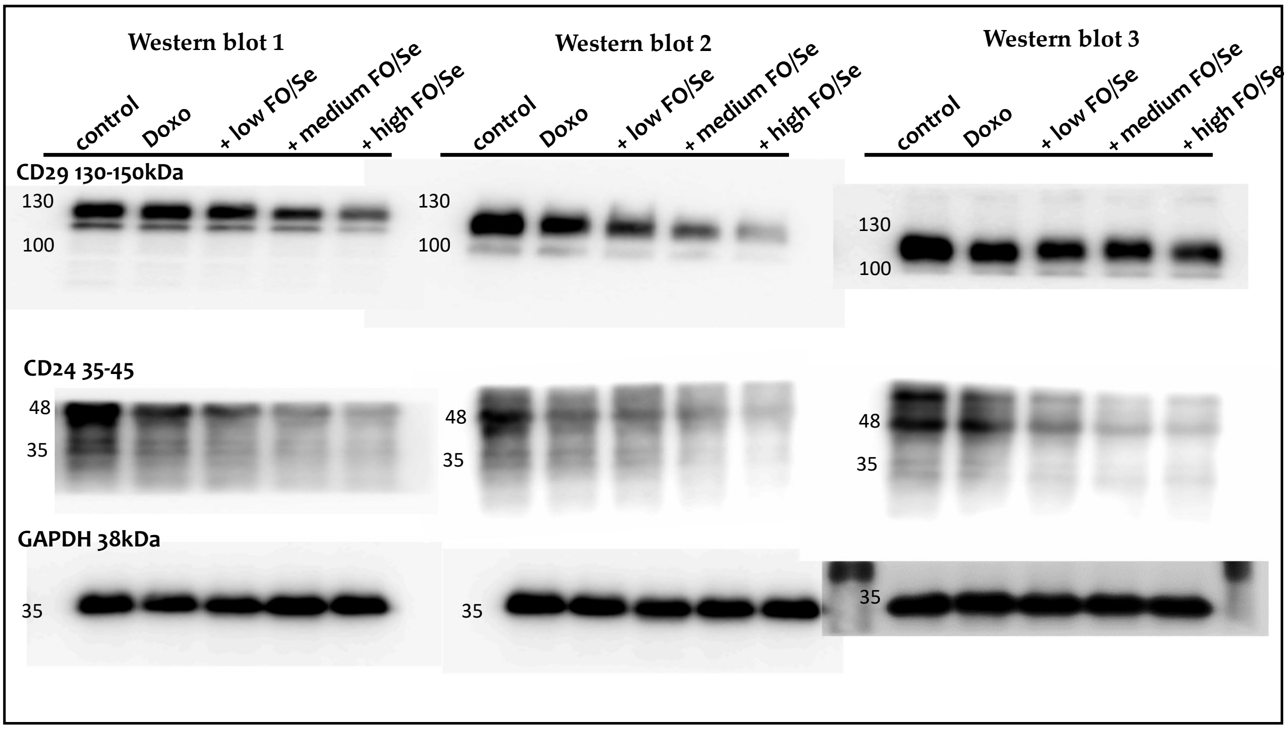
Western blot 1
Western blot 2
Western blot 3
control
Doxo
+ low FO/Se
+ medium FO/Se
+ high FO/Se
CD29 130-150kDa
130
100
CD24 35-45
48
35
GAPDH 38kDa
35

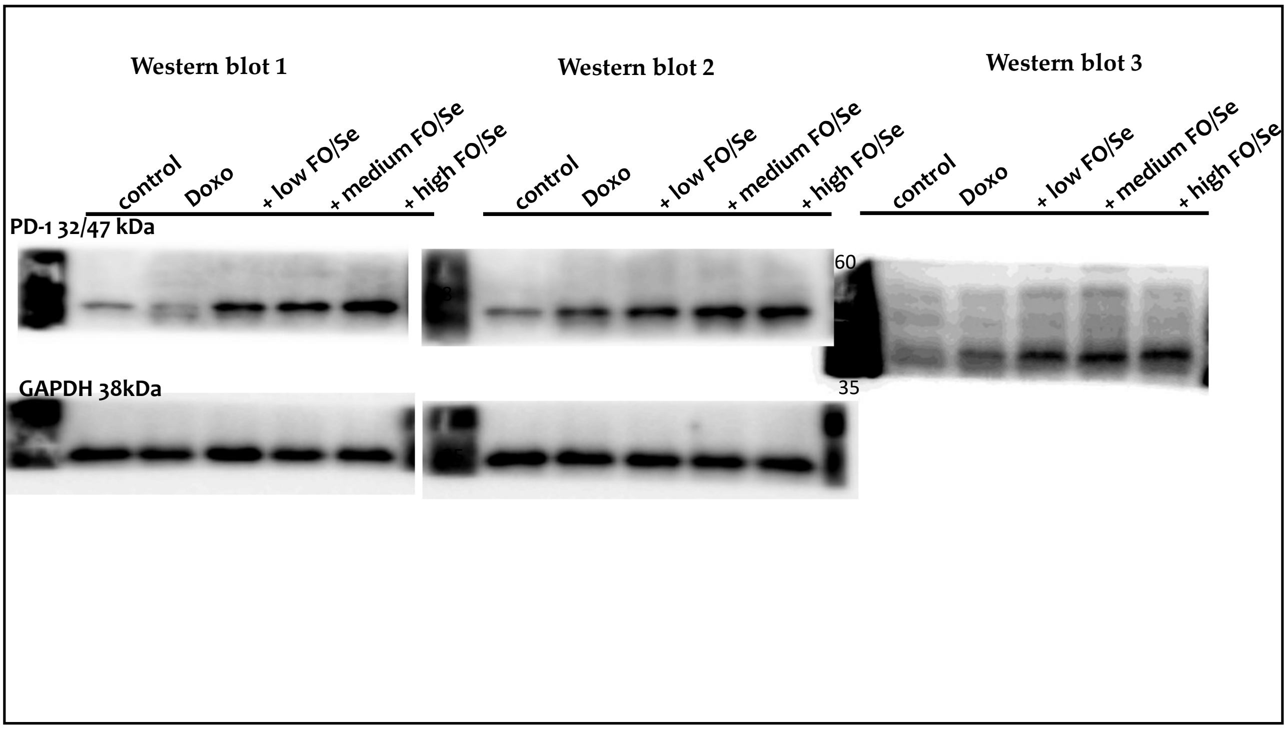
Western blot 1
Western blot 2
Western blot 3
control
Doxo
+ low FO/Se
+ medium FO/Se
+ high FO/Se
PD-1 32/47 kDa
GAPDH 38kDa
60
35

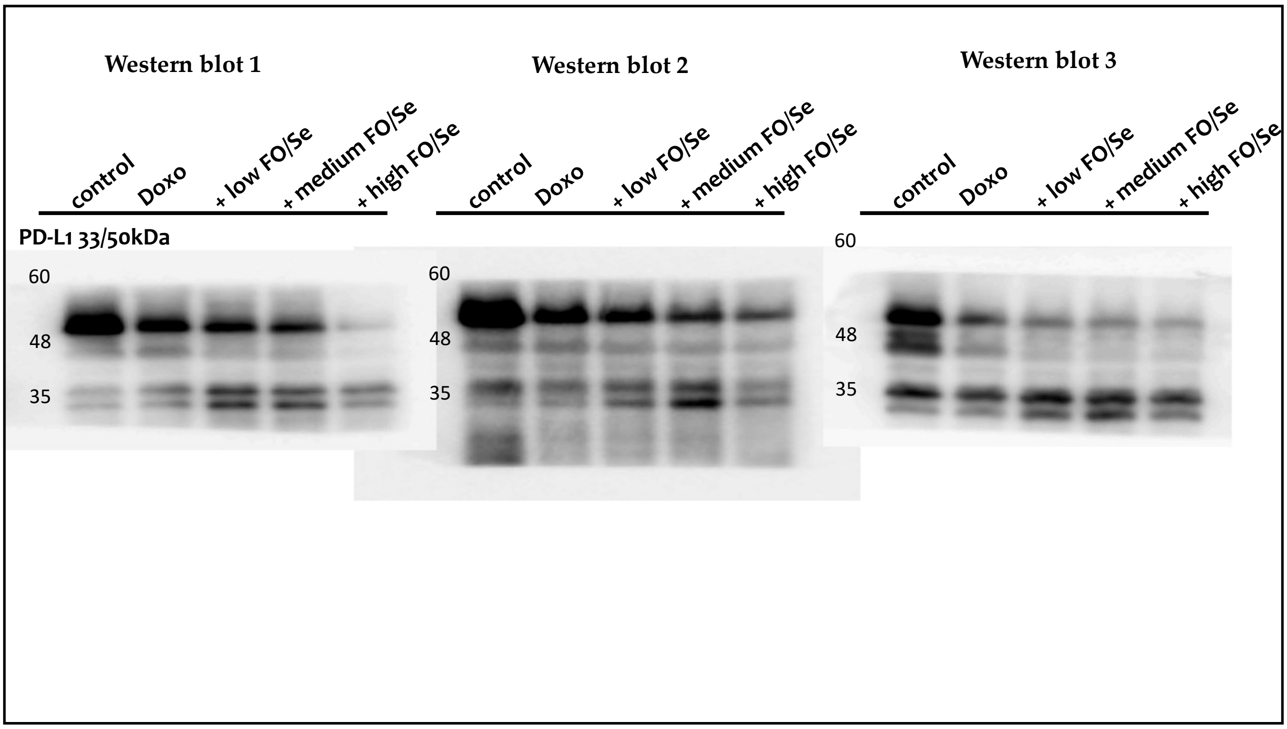
Western blot 1
Western blot 2
Western blot 3
control
Doxo
+ low FO/Se
+ medium FO/Se
+ high FO/Se
PD-L1 33/50kDa
60
48
35

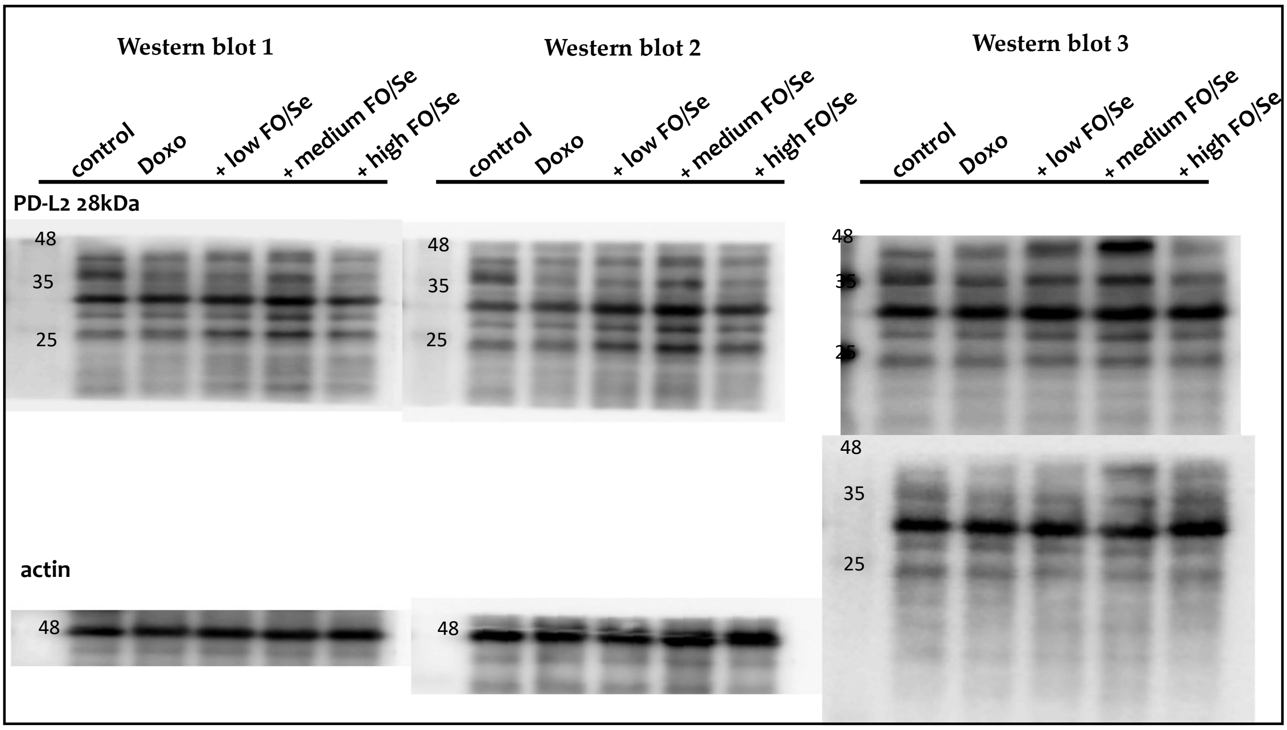

Western blot 1
Western blot 2
Western blot 3
control
Doxo
+ low FO/Se
+ medium FO/Se
+ high FO/Se
PD-L2 28kDa
48
35
25
actin

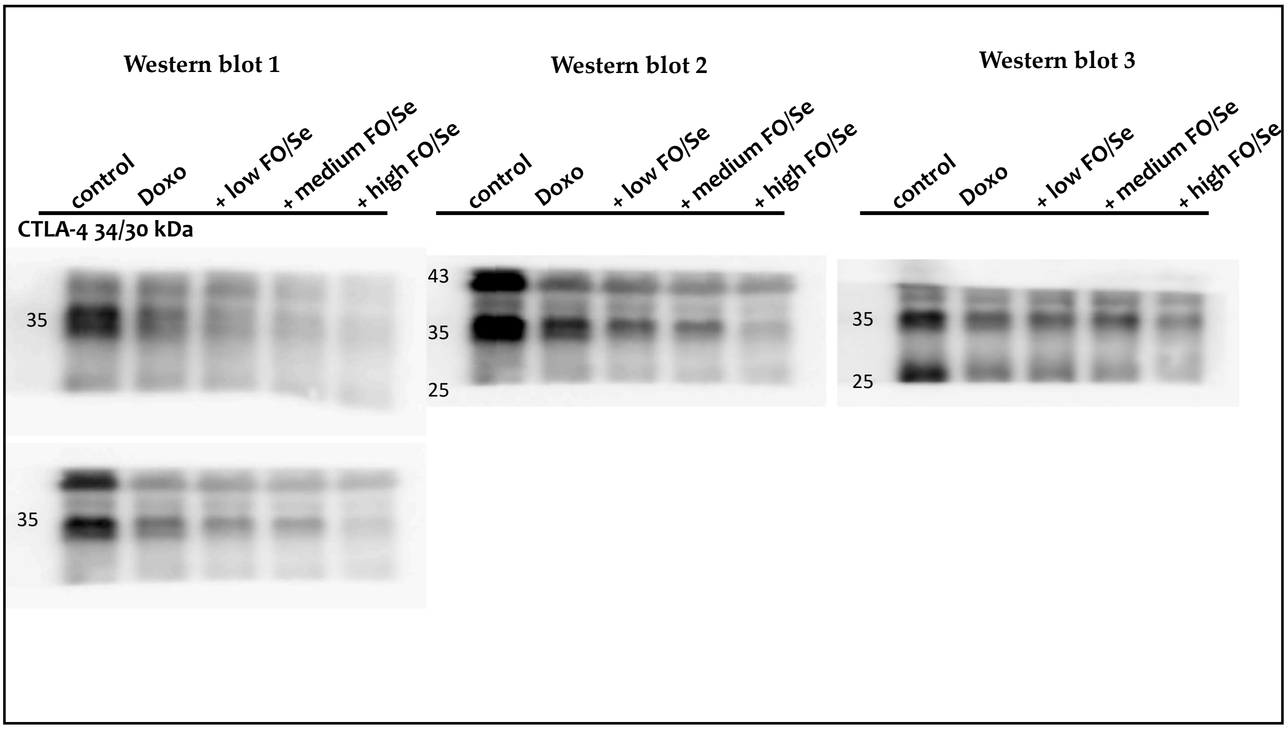

Western blot 1
Western blot 2
Western blot 3
control
Doxo
+ low FO/Se
+ medium FO/Se
+ high FO/Se
CTLA-4 34/30 kDa
43
35
25

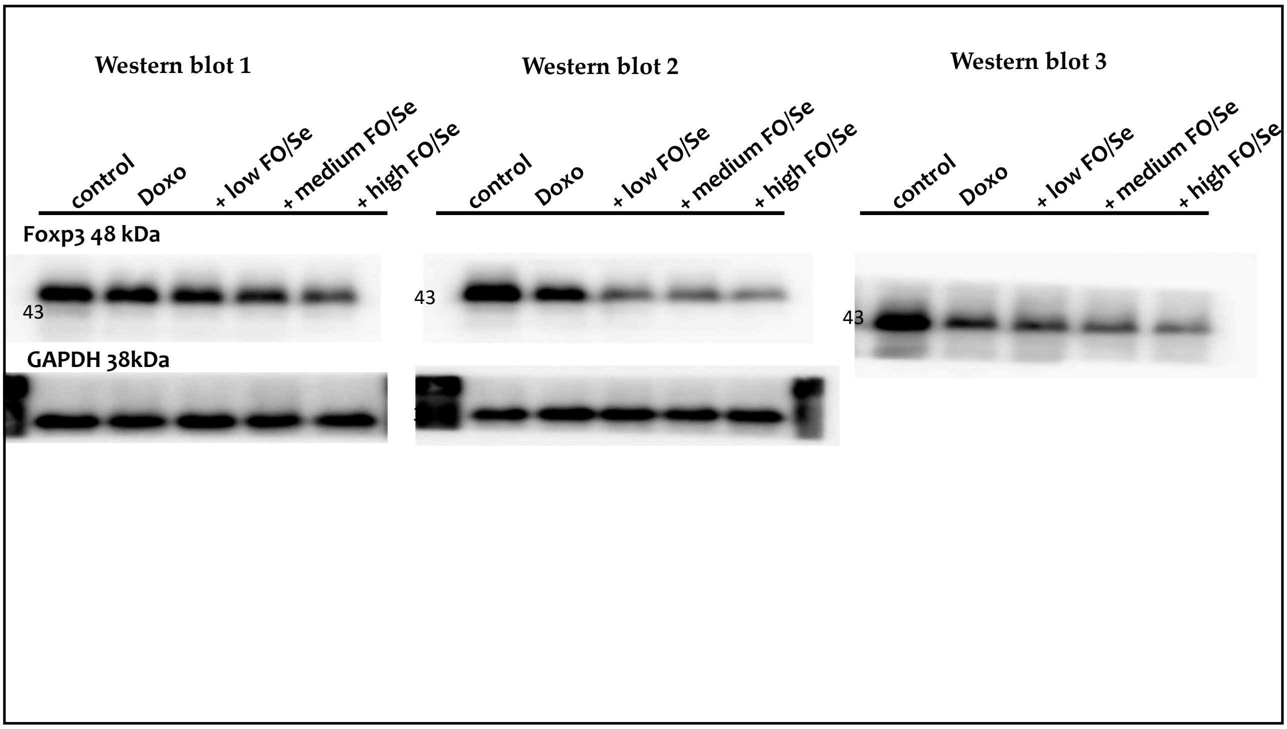
Western blot 1
Western blot 2
Western blot 3
control
Doxo
+ low FO/Se
+ medium FO/Se
+ high FO/Se
Foxp3 48 kDa
43
GAPDH 38kDa

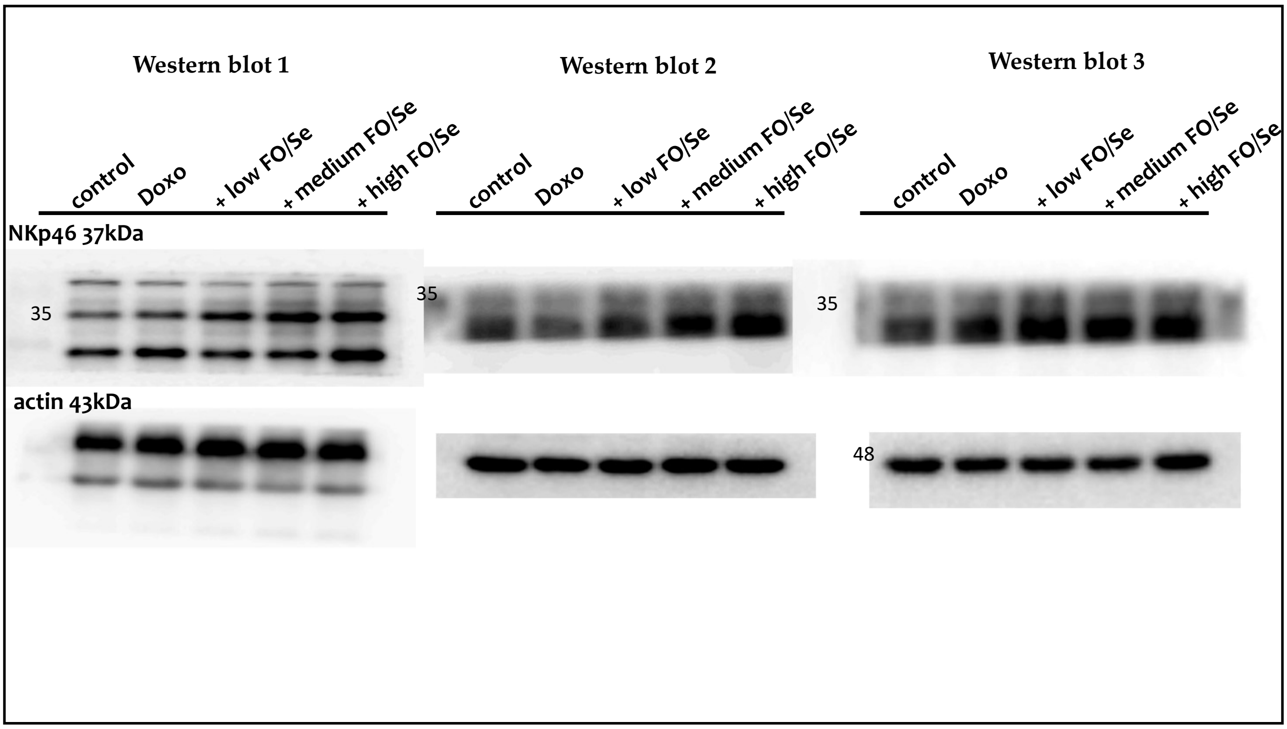
Western blot 1
Western blot 2
Western blot 3
control
Doxo
+ low FO/Se
+ medium FO/Se
+ high FO/Se
NKp46 37kDa
35
actin 43kDa
48

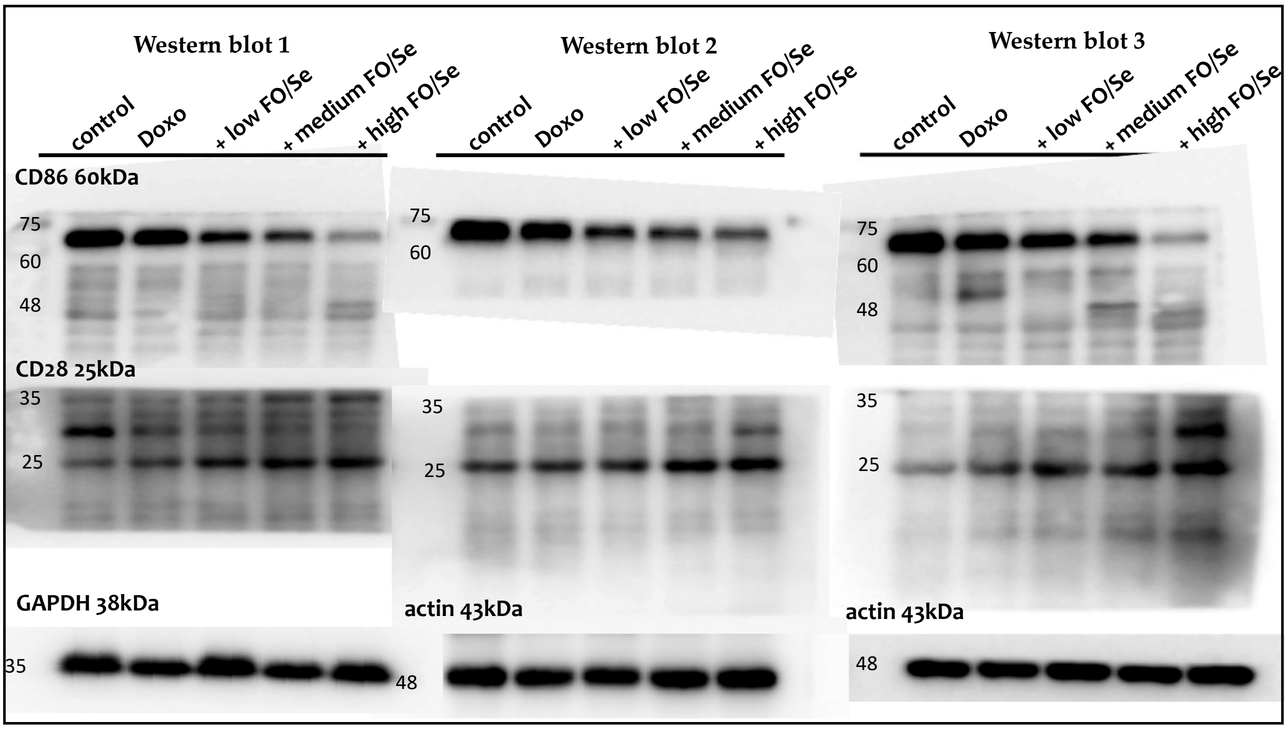
Western blot 1
Western blot 2
Western blot 3
control
Doxo
+ low FO/Se
+ medium FO/Se
+ high FO/Se
CD86 60kDa
75
60
48
CD28 25kDa
35
25
GAPDH 38kDa
35
actin 43kDa
48

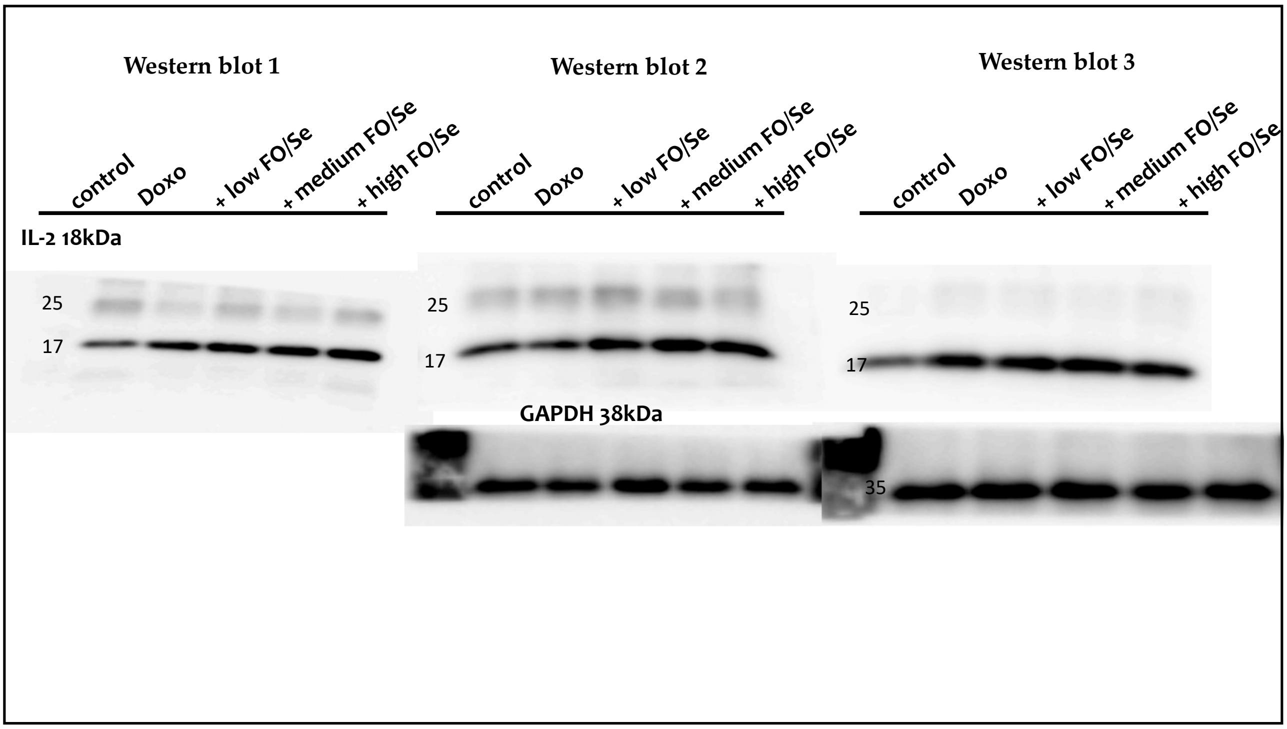
Western blot 1
Western blot 2
Western blot 3
control
Doxo
+ low FO/Se
+ medium FO/Se
+ high FO/Se
IL-2 18kDa
25
17
GAPDH 38kDa
35
